# Spirit of the English Periodicals, and Notices of English Medical Literature

**Published:** 1834-10-01

**Authors:** 


### Periscope;

OR,

### CIRCUMSPECTIVE REVIEW.

"Ore trahit quodcunque potest, atque addit acervo."

I.

### Spirit of the English Periodicals, and Notices of English Medical Literature.

### Influence of Bodily Position on the Pulse. By T. R. Blackley, A.B.

[Dublin Journal, July, 1834.]

This subject was ably investigated by Dr. Graves, in the fifth vol of the Dublin Hospital Reports, and is noticed by us in the 15th vol. of this Journal, p. 152 *et seq*. Dr. Graves acknowledged his inability to explain the reason why position of the body influenced the number of pulsations of the heart, and this acknowledgement stimulated our author to unriddle the mystery. The following extract is necessary, to shew the basis on which Mr. B. rests his conclusions.

"I believe it will be readily conceded, that the action of the heart in a strong and healthy individual, while in a state of rest, is uniform and equal, that it is possessed of a power sufficient to expel a certain quantity of blood at each contraction of the left ventricle, which power is necessary to overcome the obstacles presented to the egress of the blood.

Let us suppose, for instance, that the heart of a healthy man in the erect posture beats sixty times a minute, and at each beat expels one ounce of blood, sixty ounces per minute will be of course expelled; but if the *power* of the heart be increased or diminished, we must expect a corresponding alteration in the number of beats. Thus, if the power be *increased* one-tenth, it will require but fifty-four beats to expel sixty ounces in a minute; but if it be diminished one-tenth, it will require sixty-six beats.

To this variation of power in the heart are the phenomena of which we speak attributable, or rather, I should say, *the variation of resistance to the heart*, which comes to the same thing. A question may here be asked, namely, 'can you prove that the heart discharges a greater quantity of blood at one time than another, or that the left ventricle does not at each contraction expel the entire of its contents?'

I am fully aware that the general opinion is opposed to mine; indeed, actual experiment would seem to be against me, for it has been affirmed, that on inserting the finger into the left ventricle of a recent heart, it contracts through its *whole extent;* nevertheless, I think I can prove the reverse, from the anatomy and mechanical construction of the heart itself.

1st. There are no muscular fibres going from the substance of the left ventricle to be inserted into the opposite edges of the valves, or above them into the aorta; therefore the space immediately inferior to the valves cannot be compressed.

2dly. We never find the left ventricle perfectly closed after death, even though it should be at the time that rigidity of the muscular fibre prevails.

3dly. If the valves had no support, as by a certain quantity of blood under them, to act as a counter pressure, they would be liable to injury from the superincumbent mass of blood in the aorta.

4thly. A vacuum being formed by the expansion of the ventricle, the valves would be drawn downwards with considerable force to fill it up, and thus an additional weight be unnecessarily imposed on them.

Lastly. In a very rapid pulse, say 160, we can scarcely conceive the sides of the left ventricle to be closely approximated through their whole extent at each pulsation, and separated again, the usual quantity of blood as in health being expelled at each contraction. But we can readily admit that such a number of palpitations may be effected, the ventricle contracting but slightly at each beat; or a very small quantity of blood may be contained in the ventricle at each contraction, and thus supersede the necessity of the ventricle being much dilated; and we may observe, that in those cases where the pulse is so frequent as I have mentioned, it is never full and strong, but on the contrary, weak and thready, evidently proving that a very small quantity of blood indeed is forwarded at each contraction of the ventricle."

Our author goes on to state that, in the erect posture, the column of blood in the arch of the aorta, together with that in the carotids, presses on the semilunar valves, and opposes the egress of blood from the left ventricle. The arteries being all full, a considerable *vis à tergo* is necessary to force the blood in all directions, but especially upwards. In the perpendicular position, too, the return of blood by the veins from all the lower part of the body is much retarded by gravitation, and consequently requires greater force from the heart to drive it through the capillaries, and, if you please, pump it up from the veins.

" In the horizontal position these obstacles are lessened or removed; the blood in the carotids and arch of the aorta does not press with such force upon the valves, but chiefly the *veins*, namely, all those below the heart, being placed in the most favorable position for *spontaneously* returning their contents, remove an immense obstacle to the egress of blood from the left ventricle. Hence it follows, that resistance being opposed to the heart in the horizontal position, and the same power exerted, a greater quantity of blood is propelled at a time, and consequently the number of pulsations necessary to transmit the same quantity in a given time in the erect posture diminished.

The frequency of pulsation, then, is in a direct ratio to the obstacles presented to the heart's action, whether those be mechanical or arising from the debility of the heart itself. On the supposition of the correctness of this view, can be explained, I think, all the phenomena so closely observed by Dr. Graves."

We do not think that this explanation is correct or satisfactory. Mr. B. seems to forget that the heart, having once overcome the resistance of the arteries, and injected its contents into these vessels, their elastic and muscular coats perform all the rest. The blood is prevented from returning into the ventricle by the semilunar valves, and consequently it must be forced along the arteries and through the capillaries by the arteries themselves. Now, as far as gravitation is concerned, the arterial circulation, in all parts below the heart, is favoured rather than checked; so that the ingenious theory here broached cannot be made available. We cannot offer a satisfactory reason, ourselves, for the increased frequency of the pulse in the erect posture; but we imagine that it is far more connected with the muscular exertion of standing than with gravitation. Thus, the pulse is increased by the simple change from the horizontal to the perpendicular position; but it is still more accelerated by running up stairs, or up a hill, proving how much influence muscular contraction has on the heart and arteries. We apprehend that standing and walking give rather resistance than assistance to the arterial circulation, by the pressure of the muscles on the vessels; and the heart may thus be called upon for a greater number of contractions. The venous circulation ought to be accelerated by muscular action; and yet we invariably see the veins swell by exercise, as is very apparent in horses, and in the arms of blacksmiths and others, while straining the muscles.

---

### Danger of Ergot of Rye in large Doses.

In a report from the Wellesley Female Institution, in the July Number of our Dublin contemporary, there are a few remarks on the *indiscriminate* and *inordinate* employment of secale cornutum, by Dr. Maunsell. Mary Redmond, in the fourth month of pregnancy, was seized, after a fright and severe exercise, with hæmorrhage from the vagina, which continued at intervals for several days. On the 10th of March, 1834, at six in the afternoon, Dr. Churchill saw her, and gave her 45 drops of laudanum. At 9, she was so weak that a plug was introduced. At 7 next morning, the plug was expelled, with considerable hæmorrhage, and a grain of opium, with some acid mixture, was given every two hours. At 8, p.m. the hæmorrhage set in again, and Dr. C. gave her ʒss. of the ergot at once, repeating it in half an hour. On the 12th, she had a violent head-ache; and, in the course of the day, became delirious, and could hardly be kept in bed. These symptoms yielded to purgatives, cold lotions, blisters, &c. In a few days afterwards, the hæmorrhage again returned, and the ergot was repeated, with another recurrence of delirium, but in a slighter degree.

The second case was a Mrs. Forest, two months pregnant, who was attacked with hæmorrhage on the 21st April, and for which the usual remedies were prescribed. Next day, the discharge having recurred in an alarming degree, the infusion and substance of half a drachm of the ergot was given by Dr. Churchill in two doses, at 15 minutes' interval. After taking it, she fell asleep, and on awaking the discharge returned. Another scruple of the ergot was given her, which produced vomiting after an hour. On the following day she was in a state of half stupor, with violent headache and depressed pulse. These symptoms gave way to proper medicines. These two cases excited inquiry, when it was found that they did not stand alone. A case was related to our author by Dr. Johnson, in which, through mistake, the infusion and substance of two drachms of the ergot were given in two doses. In six hours, Dr. J. was again called, and found the patient in a state of incomplete coma, with livid face and muttering delirium. There was no uterine action induced by this extreme dose. She was delivered by the forceps, and remained 30 hours delirious and in a state of partial stupor. She had a bad recovery. In another case, where the ergot was largely used, Dr. Johnson saw complete gangrene of the external parts, with death in six hours after delivery. In a third, the same experienced physician witnessed extensive sloughing of the vagina, without any other probable cause. Mr. Cusack informed our author that similar cases had occurred in his practice. In a German work, too, the author finds that Professor Joerg, of Leipzig, made experiments on himself, his pupils, and on animals, from which it appeared that nausea, vomiting, pains in the abdomen, weight and pain in the head, with vertigo, followed large doses of the ergot.

The foregoing facts are not sufficient to prevent the moderate use of this important medicine; but they ought to be borne in mind, and prove a check to the reckless manner in which this medicine is now sometimes administered.

---

### On Scrofula. By Jas. Eager, M.D.

Dr. Eager has published a somewhat elaborate paper on the nature and treatment of a disease, which proves a great scourge in this country. The author was a house-surgeon in some of the Parisian hospitals, and had good opportunities of witnessing the effects of various modes of treatment, and especially the treatment by iodine. On the nature and causes of this too well known disease, we need not dwell. The exhibition of iodine on a large scale at the Hospital of St. Louis, in Paris, and the wonderful cures which are said to be performed there, engage our author's chief attention in this paper, and our's also.

"The use of iodine requires the greatest prudence. It has been administered internally both in a simple form and combined with iron, mercury, and potass, in the form of pill or in solution. Externally, combined with lead under the form of ointment, or dissolved in water in its simple state, either in lotion, injection, or bath. All these means have had their respective advantages. They were consequently used on various occasions. Iodine taken internally constitutes the basis of the treatment where the stomach is not affected. The results of cutaneous absorption are too doubtful to admit of frictions as the principal form of administering iodine. Children take pills with much difficulty. Pure iodine may be given in the form of tincture diluted with water. Two reasons, however, prevent us from using the tincture at this hospital. In the first place the fear of mistakes in dropping, mistakes the more easily made when there are many children under treatment; and secondly, our apprehensions lest the water of Arcueil (which is used exclusively in this establishment, and which contains a large quantity of calcareous salts,) may alter the nature of this medicine. The same objection holds good with regard to the æthereal tincture of iodine, the simple or ioduretted solution of hydriodate of potass, and the alcoholic æthereal tincture of ioduret of mercury. We use the solution of iodine in distilled water called by Lugol, "Eau minerale iodée" Each pint of water contains two grains of iodine and four grains of hydriodate of potass, the latter being added to render the former more soluble. We have not deemed it prudent to adopt the use of the solutions of different degrees of strength which Lugol employs; because in the treatment of children, particularly when they are numerous, these different proportions offer many inconveniences without any real advantage. It is much more simple to administer a solution that contains a fixed quantity of iodine, which may be prescribed at will in more or less large doses, than to have for each patient solutions that contain a variable quantity of iodine.

The dose varies with the age, the state of the digestive canal, its influence on the disease, always beginning with three ounces of the solution, which may be gradually augmented to twelve ounces in the day. This is the strongest dose we have given, viz. ℥vj. in the morning and ℥vj. in the evening. Each ounce contains the $\frac{1}{8}$ of a grain of iodine and $\frac{1}{4}$ of a grain of hydriodate of potass. In following this method we know the exact quantity prescribed. After the use of this proportion for some time, we thought to double the quantity of iodine for each pint, but were soon obliged to abandon it in consequence of the disagreeable taste of the medicine, and a sensation of heat which it occasioned in the throat. The solution may be sweetened immediately before use with sirup of gum. If mixed with this sirup long before hand, the iodine becomes decomposed, and the solution loses both its colour and taste. In order to preserve it long fit for use, it should be kept in bottles well corked and opened as seldom as possible. The smallest dose to begin with is ℥j. morning and evening, which may be increased to ℥vj. each time as before-mentioned. This dose is even stronger than that Lugol recommends for adults. When no accidents occur to contra-indicate its use, we continue this solution during four or five weeks, then we stop it, and give a purge of sulphate of soda or magnesia. The purgative is repeated two or three times before we return to the use of the solution. The suspension continues generally fifteen or twenty days. We then resume, and continue its use a month, then stop it again to give the purgative as before said. It sometimes happens that acute accidental diseases oblige us to discontinue the treatment for some time. Diarrhœa and emaciation, which have been so much dreaded, seldom occur; when they do, they always cease on stopping the medicine. I have seen but one case in which the patient became thin; all the other children, on the contrary, have become very fat. The appetite has increased, and their soft flesh become hard and coloured. I have seen a case in which the use of iodine was followed by cardialgia, and this affection ceased on using ℥j. of bark once a day, without discontinuing the iodine; and after a short time we were able to give the medicine alone. It sometimes happens, though seldom, that the use of iodine ulcerates the mouth and affects the breath as in mercurial salivations. In some females symptoms of cerebral congestion appear, such as epistaxis, which yielded to rational treatment. It is remarkable that these symptoms manifested themselves at the period of puberty, and consequently may be ascribed more rationally to a deviation of the menstrual flux, than to any other cause. Messrs. Gardner and Coindet attribute to a kind of iodine saturation, accidents which have been remarked, in some patients, such as fevers, palpitation, dry cough, tremblings, loss of strength, and swelling of the legs. At the Children's Hospital we have not remarked such cases. This may be owing to the precaution we use of purging the children from time to time. Moreover, these symptoms are extremely frequent in the third stage of phthisis in patients who never used iodine. The ioduret of iron may be given with good effect in this affection, more particularly in cases where the patients are very pale, and their flesh soft and flabby. This medicine may be given in larger doses than the hydriodate of potass. We begin by half a grain in ℥j. of water morning and evening, and increase it half a grain every four days. It may be increased to ten grains a day; in higher doses it induces vomiting. The solution of ioduret of iron produces diarrhœa much more frequently than the iodine solution. Iodine frictions are very useful auxiliaries to its action internally. We rub the tumours with an ointment composed either of ioduret of lead, ioduret of mercury, or ioduret of potassium. It sometimes happens (as with other medicines in all diseases,) that the ointment first used brings the tumour to a certain degree of diminution, and then it remains stationary. In such cases it is useful to substitute one of the others, and by this means the absorbent system is kept in a state of salutary excitement. The ointment of ioduret of mercury is composed of ℥j. axunge, and ʒss. of ioduret of mercury, that of lead of ʒj. to ℥j. of axunge, and that of ointment of hydriodate of potass with iodine, of ʒj. hydriodate and twelve grains of pure iodine to each ounce of axunge. The ointments of ioduret of mercury and potass determine a pricking pain that continues fifteen minutes, that of lead does not produce this sensation although applied to ulcers, spread on linen or charpie. A child ought to be rubbed but once a day, and an adult twice, in consequence of the skin being less irritable, and absorption less active in the latter; each friction during five or six minutes; and the quantity of the ointment varies with the size of the tumour, the minimum being that of a small pear or nut. We have in no case relied on frictions as the sole means of cure. They have always been combined with iodine internally and in baths. It is a matter of perfect indifference to which of the ointments you give a preference. For injecting fistulous trajets we use a solution of twelve grains iodine and twenty-four hydriodate of potass in one pint of water."*

An iodine bath is used in the hospital; but Dr. E. should have given us English weights and measures, instead of litres, &c., which are unintelligible to most people on this side of the Channel.

"Sixty-seven female children have been treated with iodine for a period sufficiently long to enable us to determine its effects with precision. Their age varied from four to fifteen years, a little more than half were over ten years old. In all of them the disease was of long standing; one was in the hospital since 1822, two since 1826, six since 1828, ten since 1829, twenty-six since 1830, and thirty-two were admitted since 1831, either before or after the 1st April, at which period the treat-

* Dublin Journal, July, 1834.

ment commenced. In all these children the disease presented itself under different forms. Fifteen of the sixty-seven who were treated by iodine were discharged cured of the apparent symptoms of the disease; fourteen experienced considerable amendment, which announced a speedily approaching cure. In thirteen others, although the improvement was less evident, one could judge that the cure would take place at some not far distant period; five received but little benefit from it, and, in fine, twenty did not receive the slightest advantage from the use of this medicine. Among the children cured was one admitted in 1826, two in 1829, seven in 1830, and five in 1831. Among those who experienced considerable benefit, was one admitted in 1826, two in 1828, three in 1829, five in 1830, and three in 1831. Of the twenty who were not at all relieved, two were in hospital since 1828, four since 1829, seven since 1830, and the remaining seven since 1831. All these children, with a few solitary exceptions, were much more affected at the period they commenced the use of iodine than at the time of their admission. The disease had increased with their sojourn of many months, and even years, in the wards."

Seventeen of the sixty-seven had glandular swellings. In only four of these did the tumors entirely disappear. In thirteen cases the iodine completely failed. When left to Nature, the ulcers of scrofula take many months to heal; and even then leave ugly cicatrices. "On the contrary, when art interferes, the cure is obtained in fifteen days, or a month at the farthest." Our author concludes by averring that no other remedy hitherto used in scrofula has been so successful as iodine. The paper appears to be a translation from the French rather than an original essay; nevertheless it is well worthy of perusal by practitioners in this country.

---

Anomalous Tumour in the Right Hypochondrium. By R. L. Nelson, Esq.

[Dublin Journal, July, 1834.]

The following case, which we shall greatly abridge, is very interesting.

An English physician, aged 27 years, placed himself under the care of Mr. Nelson, in July, 1833, labouring under dyspeptic symptoms of an aggravated kind. He had a delicate, bilious appearance, and stated that he had been subject from youth to occasional pain in the right side, accompanied by derangement of the bowels. He had now loss of appetite, thirst, furred tongue, inertness, slight pain in the right hypochondrium, accompanied by tenderness and fullness of the liver in this situation. Bowels confined, urine high coloured. Under slight aperients and proper diet he rapidly recovered; but returned to the narrator in a month, stating that he had hepatitis. On examination the liver was found to be much enlarged, painful to the touch, and harder than natural. He was put upon proper treatment, but improved slowly. Early in September Mr. N. discovered a small tumor below the margin of the right false ribs, apparently placed upon the inferior portion of the right lobe of the liver. It communicated to the finger a slight pulsation, which increased daily, till it became evident to the eye. At the end of three weeks the tumor was the size of half an apple. "It was accurately circumscribed, and presented both to the feel and by auscultation the peculiar purring thrill of aneurismal varix." The action of the arterial system generally became increased, with the increase of the tumor. The aorta also exhibited a remarkably loud *bruit de soufflet* through its whole course. Dysphagia was considerable. Leeches, blisters, digitalis, &c. were employed without advantage. Dr. Stokes met in consultation in the last week of September, and a careful examination was instituted and repeated several times. It was thought that there existed inflammation of the lining membrane of the aorta—enlargement of the liver—and an aneurismal tumor of uncertain nature and source. Matters grew progressively worse—the action of the heart and arteries becoming more violent, and the pulsation of the tumor more violent. In consequence of getting cold, some bronchial inflammation was set up, and three leeches to the trachea produced such a hæmorrhage as nearly destroyed life. They were obliged to give him cordials, and even quinine. In consequence of, or as a coincidence with the hæmorrhage, the aneurismal pulsation ceased altogether, and the size gradually decreased till the tumor was no larger than half a marble. Before Christmas it had totally disappeared. After this he appeared to recover—to gain strength, and even flesh, so as to be able to walk a mile without much fatigue. He was, however, rather incautious, and twice caught cold, with repetition of the bronchitis. In the mean time anasarca of the lower extremities appeared, and gradually progressed to the trunk, with difficulty of breathing and other distressing symptoms, as those of ascites and tympanitis. He died rather suddenly on the 12th of April of the present year.

*Examination post-mortem.* The liver was found to be somewhat enlarged, and generally tuberculated. The superior lobe of the left lung was hepatized—water in the cavities of the pleura—marks of inflammation in the trachea and bronchia—left side of heart hypertrophied and dilated. The arteria innominata was considerably increased in calibre, and the left subclavian slightly. The aorta, in its transverse portion, at the point where it is joined by the ductus arteriosus, exhibited a singular constriction, as if a sharp instrument had been pressed upon its upper surface until it had diminished the calibre by about one half. There was no deposition in its coats of osseous or calcareous matters, and the ductus arteriosus was open. The aorta, in the rest of its course, was somewhat smaller in calibre than usual. The semilunar valves of the aorta were either removed or completely obliterated by an irregular fleshy mass which grew from the line of junction of the vessel with the heart, and almost entirely filled the canal. The communication between the heart and the aorta was reduced to an irregular slit-like opening, through which a common probe could not pass without difficulty; and yet through it all the blood of the system found its way.

There then was the great seat of disease, all the other phenomena being mere effects, and not causes. The author very naturally asks what could have been the nature and seat of the circumscribed and pulsating tumor which occupied the attention of the medical attendants for three or four months? No trace of lesion could be detected any where to account for this tumor. We have no doubt, in our own mind, that it was a distended gall-bladder, and that the pulsation was communicated from the heart.

A case occurred in our own practice, where a circumscribed and pulsating tumor appeared in the epigastric region, and was pronounced to be aneurism of the cœliac artery by one of the most distinguished pathologists of London, and that after repeated and careful examination. After the exhibition of some doses of calomel and opium, followed by brisk purgatives, a quantity of viscid bile came away, and the cœliac aneurism disappeared. We would therefore caution our brethren against confident diagnosis in pulsating tumors; for this pulsation can be communicated, especially to a globular body filled with fluid, as the gall-bladder for example, with such astonishing similitude to an aneurism, that it is next to impossible to distinguish between them.

---

### Homœopathy.

The Rev. Thomas Everest has addressed a letter to the medical practitioners of Great Britain, in favor of the new doctrine. He commences his pamphlet with some very handsome eulogies on the medical character, and then gives us a concise history of the doctrine from its origin in the brain of a German student, in 1790, down to the present time. The Reverend Gentleman calls upon us to embrace a system which is utterly opposed to every thing that has been taught and learnt since the days of Hippocrates. "Be it right (says he) or be it wrong, it is, at any rate, utterly opposed to the present practice, and there cannot by any possibility be any alliance or truce between homœopathy and allopathy. Hahnemann's theory must either be admitted or rejected altogether. It allows of no compromise—it will not amalgamate in any way whatever with the allopathic system."

"It is doubtless well known to you, gentlemen, that all over the Continent of Europe, and in America also, Homœopathy *is said* to be flourishing—*it is said* to have performed, and to be daily performing cures which are almost miraculous; *it is said* to have so far surpassed polypharmacy, as to have cured on many different occasions dangerous fevers in 24 hours, mental affections hitherto regarded as incurable, chronic complaints of very long standing, which had defied with equal imperturbability, the lancet and the blister, the pill and the draught, the change of air, and the change of diet, the physician and the druggist, the surgeon and the nurse—it is said amongst other wonders, to have supplied a complete and almost unfailing specific against the modern opprobrium medicorum, the Asiatic cholera."

It was very right for Mr. Everest to use the saving term "it is said," for we can assure him that, as far as we have seen the practice of homœopathy in this metropolis, there is not one word of truth in all the cures that "*are said*" to have been performed by the new system. All the cures that have taken place were effected by nature, aided probably by the influence of the imagination—which has much to do in sickness. Mr. Everest calls on us to give homœopathy a fair trial, and not to condemn it untested. Our answer is, that we have seen it repeatedly tested by the homœopathists themselves, without, in any instance, the slightest proof of benefit. We ask Mr. Everest if we are justified, after such evidence, in trifling with human life, and allowing diseases to advance uncontrolled by active remedies? For our own parts we should consider ourselves culpable if we permitted the well-known resources of our art to remain in abeyance, while the inert and ridiculous remedies employed by the homœopathists were set in array against dangerous diseases. Suppose Mr. Everest were addressed by a medical practitioner, and told that, in a certain chapel in London, the gift of tongues, and inspirations of the Holy Ghost were conferred, and that this solemn truth was asserted and confirmed by hundreds and by thousands:—would not Mr. Everest have good cause for abandoning the old scriptural ritual of his church, and adopt or test the new light? We apprehend that the author of this pamphlet would spurn the idea of embracing the Regency-square system of theology, though the miracles of Irvine are not a whit more fabulous than the cures of Hahnemann.

---

SOMNAMBULISM. CASE OF JANE C. RIDER.*

We apprehend that the state of somnambulism is but little understood. It appears to us to be more allied to delirium (not from disease) than to sleep. In delirium, the mind is so occupied with one or more subjects, that it receives not, or attends not to impressions conveyed through the medium of the senses, unless these impressions relate to the subject of the delirium. But the senses, such as sight, touch, hearing, &c. are quite as alive to the subject of the delirium, and to all impressions connected with it, as if the individual were wide awake. We will endeavour to illustrate this. In very early youth we had a school-fellow who was a somnambulist. He frequently got out of bed in the night, and walked along the range, or parapet wall, of a bridge in the neighbourhood,

* By L. W. Belden, M.D. 18mo.

returning to his bed in perfect safety. The writer of this article met the somnambulist one moonlight night on his way to the bridge, and observed him attentively. His eyes were open, but he seemed to regard no surrounding object. He took no notice of his school-fellow, but kept his eye on the path along which he was walking. We followed him at some distance, in the most silent manner. He cautiously climbed up at one end of the parapet wall, and slowly marched along, apparently without fear. He got down at the other end, walked across the road, and returned by the other wall to the road again, after which he went home and to his bed. Sometimes he had an imperfect recollection of the transaction, but always asserted that it was a dream. It may be remarked that the school-boys were often in the habit of running along the parapet-walls of the bridge, by way of bravado, in their way to and from school. The boy's mother watched him one night, and when she observed him approach the bridge, she shrieked out, when the boy started wildly and fell into an epileptic fit. To these fits we believe he continued subject ever afterwards, but he ceased to be a somnambulist.

There are cases, however, which are more intricate or inexplicable than the above, and which bear a considerable resemblance to insanity, or to injuries of the brain, attended with morbid sensibility of one or more organs. In those instances where it occurs in the form of a fit or paroxysm, preceded by certain premonitory symptoms, there is, almost invariably, some disorder of a bodily function. There is entire interruption of consciousness—or rather of memory, the individual, on waking, retaining no recollection of what has passed. Knowing the fallacies which have crept in, knowingly or unconsciously, into all cases of the wonderful kind, we freely confess that we do not believe one word in ten of the most *authentic facts* that have ever been put on record in such instances. As the present company is always excepted in common conversation, so we must except from scepticism, whatever comes directly before us on the evidence of living witnesses. Some of the statements on the following case are startling; but the narrators are highly respectable, and though they may have been occasionally deceived themselves, we are quite sure they are incapable of the wish to deceive others.

*Case.* Jane Rider, aged 17 years, is the daughter of a respectable mechanic, and her mother died of disease in the brain. She was always of a mild, obliging, and intelligent disposition. Her education was much superior to her station in life. She is fond of poetry, and correct in her taste. She is plump, but rather handsome. She is subject to headaches and other symptoms of determination to the brain. She had also chorea a few years previously.

The affection in question first made its appearance in the night of the 24th June, 1833.

"I was called, under the impression that she was deranged, and such at first was my own belief. She was struggling to get out of bed, complaining very much at the same time of pain in the left side of the head. Her face was flushed, the head hot, eyes closed, and her pulse much excited. Attributing the attack to the presence of undigested food in the stomach, I gave her an active emetic, which she took voluntarily, supposing me to be her father. She rejected a large quantity of green currants, after which she became more quiet, and soon fell into a natural sleep, from which she did not wake till morning; when she was totally unconscious of every thing which had passed in the night, and could scarcely be persuaded that she had not slept quietly during the whole time."

In about a month the fit recurred; she was allowed her own way. She dressed, went down stairs, and made preparations for breakfast. She arranged the table with precision, brought cups, &c. out of a dark closet, and avoided all intervening obstacles.

"She then went into the pantry, the blinds of which were shut, and the door closed after her. She there skimmed the milk, poured the cream into one cup and the milk into another without spilling a drop. She then cut the bread, placed it regularly on the plate, and divided the slices in the middle. In fine, she went through the whole operation of preparing breakfast with as much precision as she could in open day; and this with her eyes closed, and without any light except that of one lamp which was standing in the breakfast room to enable the family to observe her operations. During the whole time she seemed to take no notice of those around her, unless they purposely stood in her way, or placed chairs or other obstacles before her, when she avoided them, with an expression of impatience at being thus disturbed."

She finally went to bed, and, in the morning, finding the breakfast things prepared, she expressed surprise and dissatisfaction that she had been allowed to sleep so long.

After this the paroxysms became more frequent, and varied without end. Sometimes the whole paroxysm was passed in bed, where she sung, talked, and repeated pieces of poetry. One night she was seen to thread a needle in the dark—another, with her eyes closed—in a third, she prepared a dinner with her eyes closed. The following is a description of the paroxysms at a period when vision was extremely acute. After this was lost the other phenomena became less remarkable.

"The state of somnambulism was usually preceded by a full, heavy, unpleasant feeling in the head—sometimes by headach, ringing in the ears, cold extremities, and an irresistible propensity to drowsiness, attended with a feeling as if weights were appended to the eye-lids. There was almost always a slight contraction of the eye-brows, the cheeks were flushed, and sometimes tinged with a crimson hue. By great exertions, the fit might be put off for hours after the appearance of these symptoms; but, in order to gain this reprieve, it was necessary for her to walk, or be engaged in some active employment. The most effectual preventive was exposure to the open air. The moment these precautions were relaxed and sometimes even in the midst of her active duties, she experienced what she described as a sense of rushing to the head, attended with a loss of the power of speech and motion. If in this state she was immediately carried into the open air, the fit was often arrested; but if this was delayed a moment too long, she lost all recollection, and could not by any efforts be aroused. To a spectator she appeared like a person going quietly to sleep. Her eyes were closed, the respirations became long and deep, her attitude, and the motions of her head, resembled those of a person in a profound slumber. During the fit, the breathing, though sometimes natural, was often hurried, and attended with a peculiar moaning sound, indicative of suffering. At times the pulse was accelerated, but generally it did not vary much from the natural standard. I have remarked, that in her first paroxysm the head was hot, but such was not commonly the case, nor was there any peculiar throbbing of the temporal arteries—the hands and feet, however, were almost invariably cold.

Her manner differed exceedingly in different paroxysms. Sometimes she engaged in her usual occupations, and then her motions were remarkably quick and impetuous—she moved with astonishing rapidity, and accomplished whatever she attempted with a celerity of which she is utterly incapable in her natural state. She frequently sat in a rocking-chair, at times nodding, and then moving her head from side to side with a kind of nervous uneasiness, the hand and fingers being at the same time affected with a sort of involuntary motion. In the intervals of reading or talking, and even when engaged in these very acts, her nods, the expressions of her countenance, and her apparent insensibility to surrounding objects forced upon the mind the conviction that she was asleep. Occasionally she was cheerful, disposed to talk, and willing to exercise her powers; the greater part of the time she was irritable and petulant. Pain in a circumscribed spot on the left side of the head was, I believe, always an attendant on the paroxysm, and frequently occasioned a degree of suffering almost beyond endurance. To this spot she invariably pointed as the seat of her agony when she repeated the expression, 'it ought to be cut open, it ought to be cut open.' Occasionally the whole system was thrown into agitation, and she presented the appearance of a person in a violent fit of hysterics.

Her eyes were generally closed, but at times they were stretched widely open, and the pupil was then very considerably dilated. These different states of the eye seemed to occasion no difference in the power of seeing—she saw apparently as well when they were closed, as she did when they were open. In the day time she always had the eyes covered with a bandage during the paroxysm, nor would she allow it to be removed for a single moment, unless the room was unusually dark. In order to test the sensibility of the eye, I took one evening a small concave mirror, and held it so that the rays proceeding from a lamp were reflected upon her closed eyelid. When the light was so diffused that the outline of the illuminated space could scarcely be distinguished, it caused, the moment it fell on the eyelid, a shock equal to that produced by an electric battery, followed by the exclamation, 'why do you wish to shoot me in the eyes?' This experiment was repeated several times, and was always attended with the same result. It was also tried when she was awake, and the effect, though less striking, was very perceptible. The same degree of light thrown on my eyelids, occasioned no pain.

How far she was sensible to the presence of surrounding objects, it is very difficult to determine; indeed facts seem to prove that she was not, in every paroxysm, alike in this respect. In the early stage of her complaint, she appeared to take little notice of persons, unless they were connected with her train of thought, and then she regarded those with her only as the representatives of the persons whom she imagined to be present. Nor did the sight or the hearing have any tendency to correct the false impression. Thus, in her first paroxysm, she regarded me as her father, and continued to do so as long as I remained with her; but, in her subsequent fits, this idea was never revived. Her conception of persons was generally made to correspond with the idea of the place in which she conceived herself to be. She was in the habit, when well, of spending her evenings in the room with the children of the family, and it was in their company that she often imagined herself to be during the paroxysm. The questions which were at these times proposed to her to test her powers of vision, were cheerfully and readily answered, because they were questions which it was natural for children to ask; or, at least, she supposed them to proceed from children. Much that she said was also directed to them, though it was evident, at times, her conceptions and perceptions were strangely intermingled. In a paroxysm, soon after the arrival of her father, he asked her a question which she answered by addressing a little boy belonging to the family, who was not then in the room; but his knife which he placed in her hand, she immediately recognized as her father's, and wondered how that came to be in Springfield while he was in Brattleborough. At a later period of her complaint, she appeared to comprehend more of what transpired in her presence, and accordingly she obstinately refused to read cards, or submit to experiments of any kind. These trials she then evidently regarded as so many attempts to impose upon her; and in adopting this conclusion she reasoned with perfect consistency; for if she actually could see as she appeared to—if to her vision, night was converted into day, and darkness into light, while she was unconscious of any thing peculiar to herself, what could be more annoying than to be constantly teased with questions which to her senses were perfectly obvious? If a request were made of her which appeared reasonable, especially if it related to her customary duties, she readily did whatever was required."

It is curious but certain that she recollected, during a paroxysm, circumstances that had occurred in a previous attack, but which had been forgotten in the interval. Proofs of the fact are adduced, but we need not quote them. All attempts to rouse her from these states of somnambulism were fruitless. She heard, felt, and saw, but the impressions would not awake her. A pailful of cold water was poured over her in one instance, when she exclaimed, "why do you wish to drown me." She then went to her chamber, changed her dress, and returned. Large doses of laudanum were given her to relieve pain, which it did, and she awoke much sooner afterwards. At the termination of a paroxysm, she always sunk into a sound natural sleep. The paroxysm appeared to be connected with disordered digestion. Though the appetite was good, the food occasioned oppression, with headache and acidity.

In November, experiments were made to ascertain the acuteness of her vision. The following will suffice as an example.

"She was seated in a corner of the room, the lights were placed at a distance from her, and so screened as to leave her in almost entire darkness. In this situation she read with ease a great number of cards which were presented to her, some of which were written with a pencil, and so obscurely, that in a faint light no trace could be discerned by common eyes. She told the date of coins, even when the figures were nearly obliterated. A visitor handed her a letter, with the request that she would read the motto on the seal, which she readily did, although several persons present had been unable to decipher it with the aid of a lamp. The whole of this time the eyes were to all appearance, perfectly closed."

As her malady was aggravated by the curiosity of visitors, and the experiments made on her, she was removed to the hospital in Worcester (America) on the 5th December last. Here Dr. Woodward kept an exact register of occurrences corroborating, in all essential points the foregoing statements and many others. In the hospital she was put on a regular system of diet and medicine—her health greatly improved—and her somnambulism diminished. When the accounts closed she was a great deal better, and we have reason to think she will completely recover. From various circumstances detailed in the narrative, but which we could not introduce here, we are strongly impressed with the belief that there is no imposture in this remarkable case. We are certainly not much inclined to credulity, and are much more likely to run into the opposite extreme; but we see nothing in the foregoing case, extraordinary as it may appear, which is incompatible with the powers of the human frame, and its spiritual tenement under peculiar states of morbid excitement.

---

### NITRATE OF SILVER IN EPIDEMIC CHOLERA. By CHARLES LEVER, M.D.

We should not have noticed this subject, were it merely as setting forth a remedy for a disease which lately frightened these isles from their propriety. But it is on another account, namely, the safety with which the nitrate of silver may be taken internally, and that in large doses. The name of the medicine, and its caustic effect on the external surface of the body, have checked its internal use; though the phenomena attendant on its application to sores and morbid stuctures generally, might have lessened the fears of practitioners rather than increased them. The nitrate of silver is applied to an irritable ulcer on the leg or other part, and the pain is very little, while the irritability is seen to be materially lessened in a short time afterwards. To give a still more striking example: the fauces are red and sub-inflamed—the passage of food gives much discomfort; and there is a constant secretion of viscid saliva from the parts, clearly the product of inflammatory action. A few applications by means of a sponge to the fauces relieve or cure all these symptoms. Now it is to be remembered that the membranes at the extremities of all tubes are much more sensitive than any other portions of the canals; and therefore, as we find that the fauces, the pharynx, &c. will easily bear a solution of nitrate of silver containing five or six grains to the ounce, why should we fear that the stomach itself would be unable to bear a similar medicine? We have exhibited the nitrate of silver to some hundreds of patients—often in the dose of four and six grains a day, and never saw any injurious consequences result. The remedy is exceedingly valuable in many affections of the stomach.

In our Liverpool contemporary of July last, Dr. Lever has stated that during extensive observation of cholera in the cholera hospitals of the north of Ireland, he was led to attribute the general failure of remedies to the great irritability of the stomach, which prevented the reception, or the retention of medicines. The first case which Dr. L. relates was one of the most hopeless and deplorable character. The face, neck, and chest were deeply livid—skin cold—fingers corrugated—hearing gone—voice inaudible—pulse imperceptible—thirst insatiable; but vomiting incessant, whenever any thing was taken into the stomach. In this forlorn condition, Dr. L. dissolved 30 grains of the nitrate of silver in three ounces of distilled water, which she swallowed at once. She lay quiet for six minutes, when she vomited up a small quantity of whitish turbid fluid, and then remained perfectly at rest for twenty minutes, when Dr. L. left her. In three hours he returned, and found that the patient had sunk into a kind of slumber, and had had no recurrence of vomiting. The other symptoms remained the same. She continued in this state till the next day, without sickness; but with intense heat in the stomach. She drank freely all the night of weak brandy and water, without rejecting any of it. Reaction took place, and she completely recovered. Dr. L. saw the patient for nineteen months afterwards, and she never evinced any symptom of gastritis or other affection of the stomach. In the second case 20 grains of the nitrate were given in an ounce of distilled water. Most of it soon came up; but the vomiting ceased, and recovery took place. The usual dose, however, was ten grains to the ounce of distilled water. We think this document valuable, not only as respects the treatment of cholera; but as proving that the remedy, even in large doses, is unattended by danger.

---

Observations on Internal Aneurisms. By Professor Harrison, of Dublin.

The following observations are contained in a letter from Professor Harrison to Dr. Stokes, in the July Number of our Dublin contemporary.

"In alluding to the pain, among other symptoms attending internal aneurisms, I have remarked, that although it is very frequently stated that the degree of suffering attending thoracic aneurism is trivial, when compared with that complained of in a similar lesion of the abdominal aorta; yet exceptions not unfrequently occur. Thus, I have known cases of aneurism of the arch of the aorta to have been accompanied almost through their entire course with intense, though often intermitting pain in the region of the heart, in the spine, or through the chest generally, or in one or both arms; while, on the other hand, I have seen instances of this disease in which the suffering was so trivial, and the symptoms were so faintly and equivocally expressed, that the disease was not ascertained until the *post mortem* examination revealed its real nature. In forming our diagnosis, then, in suspected cases of thoracic or abdominal aneurism, too much importance or reliance is not to be placed on the mere circumstance of pain being either slight or absent, or intense and of almost continued duration. The change in a blood-vessel denominated aneurism is of slow progress, and unattended by acute inflammation; the arterial tissue, though peculiarly organized, is not very sensible to pain, or subject to acute disease, therefore aneurism is not painful *per se* but only in proportion as the tumor may interfere with some adjacent organ, or excite irritation in some contiguous sensitive structure. Thus aneurism in the arch of the aorta may disturb the action of the heart, or may irritate the trachea, or by extending, compressing, or in any way disarranging the neighbouring nerves, such as the left recurrent, the phrenic, or the cardiac plexus, may give rise to severe paroxysms of pain, both local and remote; yet in other instances of disease in nearly the same situation, the tumour may so shape its course as to steer clear of any interference with surrounding objects, and thus avoid all excitement or irritation, or even interruption, to their functions, except so far as the latter may be impaired by the anormal condition of a vessel whose integrity must be so very essential to the existence and well being of the economy at large. The same remark may apply to aneurism of the thoracic aorta in the posterior mediastinum; the tumour in some cases proceeds through all its stages to its fatal termination, without much accompanying local pain, or decided premonitory indications, whereas in other cases, acute spinal irritation, difficult and painful respiration and deglutition, are almost constant concomitants; attention to the anatomical connexions of the vessel in this situation, and to the different directions it may take in different instances, may explain the variety of symptoms that present in different cases.

The abdominal aorta is in connexion with several important organs through its whole course; it is also invested in *three-fourths* of its circumference with a plexus of nerves, branches of which extend along the different arteries to the several viscera. All the visceral arteries are remarkable in this respect, but more particularly the renal, gastric, and hepatic; one continued nervous network surrounds each from its origin to its final distribution. From these circumstances, we should expect that aneurism of the abdominal aorta, or of its branches, should be attended with much pain and functional derangement; and such is generally, but not uniformly, the case. I have witnessed the dissection of three cases of large aneurism which had burst behind the peritoneum, and which, during life, had not been attended with much pain, or with any decided symptoms which could lead to a positive diagnosis. In each of these the tumour was connected with the posterior part of the artery; in one case it was situated close to the diaphragm, and the blood was partly injected into the posterior mediastinum, and partly into the abdomen, about the pancreas; in the second case, the tumour had burst into the cellular tissue round the kidney; and in the third case, the symptoms had been during life so similar to those of psoas abscess, that uncertainty as to the true nature of the disease occasionally existed— (See Surg. Anat. of the Arteries, vol. ii. p. 25.) Although I have not sufficient facts before my mind on which to groud the assertion, I should yet consider that when aneurism arises from the *forepart* of the abdominal aorta and extends into the cavity, pain, and more or less disturbance of the adjacent viscera, will be more likely to occur. The stomach I have remarked to sympathise extremely in these affections; hæmatemesis has frequently occurred during their progress, and in one case death immediately followed a copious vomiting of blood, which must have been supplied from the capillaries of this viscus, as the aneurismal sac had no communication with it, and had poured its contents in a totally opposite direction.

In speaking of abdominal aneurism, I am led to another remark which appears to me to present some interest, namely, that in the abdomen we frequently meet with aneurisms of the *smaller* arteries, not merely the cœliac axis, or the mesenteric, but of the gastric, splenic, hepatic, &c. I have seen examples of each of these. I also recollect a remarkable case, in which, on opening the abdomen, I was surprised at the livid colour and great size of the omentum; it was injected with grumous blood, from the arch of the stomach to near the lower border of this process. On careful examination I found it had proceeded from an aneurism of the left gastro-epiploic artery; the tumour was about the size of an egg, and had given way by a sort of slough between the layers of the gastro-colic omentum; the surrounding parts were slightly thickened, but the stomach was free from disease, as was the peritoneum from inflammation. In the Museum of the College of Surgeons there is a specimen of aneurism of the coronary artery of the stomach. I have seen one case of aneurism of the right hepatic artery, including the root of the cystic; the tumour had not opened; the patient died of dropsy and disease of the heart; the gall-bladder was shrivelled and empty. I have dissected a case of large splenic aneurism which caused death by bursting behind the peritoneum: the spleen was unusually small. Many years ago I saw an aneurism of the right spermatic artery about an inch distant from the aorta; the testicle was of the usual size, and apparently healthy; the tumour was about the size of a nut, and had not caused any injury to the surrounding parts, though it is probable, had the individual lived for some time, it would have proved fatal by bursting. In the course of last winter, I accidentally found in the dissecting room an aneurismal tumour on the left renal capsular artery; the subject was a female, about eight or ten years of age; the tumour was solid to the feel, and on cutting into it was found filled with successive laminæ of fibrinous matter; very little cavity remained, so that the disease might be considered as undergoing a natural or spontaneous cure. Although aneurism is a disease common to all parts of the vascular system, yet it is certainly very seldom found in any of the external arteries, except the principal trunks, such as the carotid, axillary, iliac, femoral, or poplitæal. I do not recollect any case of spontaneous aneurism in any of their smaller branches, though such are not unfrequent consequences of wounds or injuries; even in an artery of the size of the brachial, spontaneous aneurism is very rare. In the cranium it has been met with in the internal carotid, and in some of its primary branches; it has been also found, but very rarely, in the coronary arteries of the heart. It might prove an interesting inquiry to consider why the small arteries of the abdomen more frequently present this disease than arteries of a similar size do in any other situation. Is the cause of this fact to be found in any peculiarity of structure, of course, of connexion, or in function? probably on each of these circumstances it more or less depends. As to structure, the abdominal arteries are certainly very weak, particularly their middle coat; hence their great liability to rupture in the common process of injecting the dead body. As to course and connexion, no vessels in the body are more remarkable for numerous turns, angles, flexuosities, &c., and most of them run very much unsupported, except by the general pressure of the parietes of the abdomen. In function, too, many peculiarities present themselves to our attention which may serve to explain the frequent occurrence of this disease in the abdomen, as well as the great liability of the mucous surface of the alimentary canal to severe and frequent hemorrhage: thus, the quantity of the blood circulating through this cavity must be very considerable; the active function that is so *frequently* exercised in *different* parts of the apparatus must require a proportional supply of blood, so that increased vascular actions must constantly occur in different situations and in rapid succession. The very remarkable freedom of anastomosis between the several vessels may probably be designed to facilitate the more rapid course of the blood from one organ to another, according as the respective functions of either may require it. The irritability of the small vessels may be inferred from the great supply of nerves with which they are furnished. I have not sufficient facts before me to decide on the pathology of these small abdominal aneurisms. I have not observed any diseased condition of the coats of the vessel in the vicinity of the tumour, so commonly observed in thoracic aneurisms. From the observations I have made, which, however, are insufficient, I consider that they commence by rupture, and not by dilatation of the internal tunics.

Yours very truly,

ROBERT HARRISON."

In a commentary on the foregoing paper, Dr, Stokes applauds the ingenious explanation of Professor H. in respect to the comparative frequency of aneurisms of the internal and external vessels—namely, the sympathies of arteries with organs. This leads Dr. Stokes to notice a species of arterial throbbing, either in the abdominal aorta itself, or, as Dr. Houston supposes, in the second order of vessels. The nature of arterial throbbing is little understood. It has been described as a nervous phenomenon—as the result of pressure on the vessel—and as a kind of aortitis.

"But there is a pulsation of the abdominal aorta or its immediate vessels, which is symptomatic of inflammatory disease in the digestive system, and which a long experience enables me to say may be considered an important assistance in diagnosis. A throbbing generally commensurate with the disease; removed by treatment calculated to relieve enteric inflammation, and aggravated by every thing which will increase this affection. In other words, we may have from enteritis or peritonitis a throbbing of the abdominal aorta or its vessels, perfectly analogous to the morbid action of the radial artery in whitlow, or of the carotids or temporal arteries in cerebritis."

The cases in which Dr. S. has most frequently observed it, are those of the gastro-enteric fevers of Ireland. Considering the latency or obscurity in which these fevers often exist, Dr. S. considers this means of diagnosis as a very important one. He does not mean to contend that this pulsation exists in all cases of gastro-enteric fever; but in many. It occurred also after corrosive poisons had been swallowed, and where the pulse was extinct at the wrist.

"In these cases we have frequently the following group of circumstances: fever, prostration, thirst, tenderness of the epigastrium or the ileo-cœcal region. The pulse at the wrist is often small and feeble, while the abdominal pulsations are comparatively violent. In most cases the other symptoms of gastro-intestinal disease are sufficiently plain. But in several instances this want of proportion between the action of the radial and abdominal arteries, *combined with fever*, has been the principal indication of enteric disease."

In some instances, he found that the pulsation was strongest in the direction of the right iliac artery, and subsided with the symptoms of the gastro-enterite. He has seen it with and without the occurrence of diarrhœa, and frequently re-appearing with relapse, errors of diet, or hypercatharsis. These circumstances, he thinks, may throw some light on the supposed cures of aneurism according to Valsalva's plan of treatment.

Our own experience entirely coincides with that of Dr. Stokes, in respect to epigastric pulsation—excepting that we are quite sure of having seen it arise from gastro-enteric irritation, as well as inflammation. We have seen it removed by tonics and sedatives. But the symptoms of irritation and inflammation assimilate so closely together, that it is often exceedingly difficult to draw the line of demarcation.

---

ON MANUAL ASSISTANCE IN ABORTION. By Mr. WAINWRIGHT, of Liverpool.

Mr. W. has published a paper on this subject in the 3d Number of the Liverpool Medical Journal, which deserves attention. Mr. W. does not advocate indiscriminate interference in cases of abortion; because, under proper treatment, the majority of cases will require no manual assistance. He properly observes, also, that "until every hope is lost of preserving the ovum, or until the condition of the parent demands energetic assistance, the adoption of manual aid would be altogether unjustifiable." Mr. W. contends that, in all severe cases of abortion, the state of the os uteri should be ascertained, and that as early, and as effectually, as during actual labour. This examination is peremptorily required when the discharge goes on from day to day, confining the patient to bed, and blanching her countenance. The following appear to be the practical maxims laid down by our author.

1st. "In many instances, *a separation and partial expulsion of the ovum* will be found: and very slight assistance, by moving it from side to side, and gently drawing forwards, will at once remove the whole from the orifice, and the hæmorrhage will immediately cease. Case No. 2 offers an example of what may be experienced under these circumstances. The assistance required in this case was certainly not slight, nor easily afforded, yet the end was fully attained, and no mischief occasioned. It will be observed, that I found it requisite to steady the uterus whilst I introduced the fingers through the orifice, and that this was effected by the left hand applied above the os pubis. I must add, however, that I do not think this plan will be effectual in every case, as in some individuals I have found it almost impossible to overcome the action of the abdominal muscles, especially of the *recti*. These seem to act, in some cases, almost involuntarily, when the hand is applied to the abdomen, whilst in others, the hand may be forced with little difficulty into the pelvis. The vagina, too, will offer some difficulty occasionally, and doubtless more when the patient has never borne children. It is unnecessary, however, to dwell upon this point, as it must be evident that unless the hand be allowed some degree of liberty in its movements, the operation in question cannot be effected.

2dly. The next two states I would allude to, are, first, where the *os uteri is found dilated, or dilatable, and a body is found projecting above;* and secondly, where the *os uteri is found dilating, and the membranes projecting;* especially during the continuance of a pain. These cases I believe to be, in many instances, the same, only in a different stage. I say, in many instances, because I am quite aware how often the tender membranes give way, allowing part of the conception to be expelled, whilst the residue remains. I class them together, however, because the same treatment is often required. Should the symptoms not be particularly urgent, and especially if the pains be frequent, it may be well to wait a little, that, in the first case, the contents may be propelled somewhat lower, and that in both, if possible, the natural efforts may accomplish the expulsion. Under other circumstances, however,—as Cases No. 3 and 4 will shew,—manual assistance may be effectively and safely given.

3dly. It will sometimes be found, on enquiry, that a *fœtus has been expelled, but no secundines have been discovered.* Of course a most diligent search will be made for these, as it is very possible they may have been overlooked, or mistaken for a coagulum. Should the search, however, be in vain, I could not acquit the practitioner of neglect, did he omit a vaginal examination, and were his patient afterwards to suffer from any of the effects of retained placenta. The decidua will often be found hanging at the os uteri; or if the pregnancy be rather more advanced, say about four months, it is not improbable that the funis,—or some apology for it, or a shred of membrane, or something,—may be suspended at the vagina, or at the os internum, and then the existence of something more within the uterus may well be inferred. Case No. 1 was of this description, and the treatment I adopted then I firmly believe to be the best that could be employed. The patient recovered extremely well. Some time had elapsed before I arrived, and I doubt not this explains the contracted state of the parts and the difficulty I had in extracting the portion which remained. I think, therefore, as little time should be lost as possible; and that a gentle and cautious effort should be made to dilate the os uteri, and to extract that which remains. In the later periods, at least, it may be remembered that the orifice has once been dilated to admit the passage of the fœtus, and surely little injury can be inflicted by distending it to the same extent, and often little more will be required. Here I would remark, that much misapprehension has been caused by this observation, 'that as before the 6th month the *hand* cannot be introduced into the uterus, therefore no manual aid can be given before that period.' Now the truth is, that in the earlier months it is absurd to think even of introducing the hand. One, two, or three fingers are all that are required, and that are almost ever spoken of. Case No. 6 exhibits the fatal effects of allowing the placenta to remain. It is true, that in this case the first gush of blood might be sufficient to account for all the consequences, yet the subsequent losses must have contributed to the fatal result.

4thly. At other times, however, whether a portion of the embryo be expelled or not, *a rigid state of the os uteri* will be found, absolutely defying the most resolute to overcome it. One author, I find, confesses he could not succeed, though 'he tried with all his might, and repeated his efforts ten times!' This, it may be presumed, would oftener be found in first pregnancies, and here, I hope, no one will follow the example of this courageous practitioner. I may add, for the credit of modern obstetricy, it was not a modern accoucheur. This, then, is the case of all others for the tampon. I have never met with this case, and I must rely, therefore, on the testimony of others. It is on this also that I rely, when I suggest, during the progress of such a case, the propriety of occasionally ascertaining the state of the os uteri. It may not be dilated at one visit, whilst it may be amply so at the next, and might then admit of manual aid.

Lamotte was called at two in the morning to an abortion of five or six months, from accident, attended with severe flooding. The os uteri was very rigid, but at six it was sufficiently dilated, and he effected the delivery. Giffard relates the case of a woman at about her sixth month, who had flooded for two or three days before he saw her. On the first day of his visiting her, he found 'the os internum not dilated enough to admit the end of one finger, and not easily to be dilated; on the second day she grew weaker, but still he could not dilate the orifice, but on the third day it was in a different state, and he quickly delivered her, when the hæmorrhage immediately stopped.' The same author relates an interesting case (No. 153), advanced only to the ninth or tenth week. 'The woman had had hæmorrhage for some days, so that she was very much sunk, and had fainted several times. The os internum was very narrow, not allowing one finger to pass up; yet with the end of one finger he felt a soft substance within, lying at the mouth of the womb. She had much pain in the back. Three hours after, the orifice admitted one finger, which he passed directly through, and, bending its extremity, hooked out the contents, when all pain and flooding ceased.'

The above quotations, I think, will be admitted as warranting the practice I have suggested under this head, and may also be taken as strongly illustrative of the general question.

5thly. The above cases I supposed complicated with hæmorrhage. There is, however, another class of cases, unaccompanied with this alarming symptom, in which I believe manual aid may most advantageously be afforded. I allude to those in which the fœtus has been expelled with *little or no flooding, but where the secundines (decidua or placenta) remain.* Cases No. 5 and 7 are instructive cases in point. The treatment adopted by Mr. Park fully shews the practice usually employed, and this, with his observations attached, may, in the opinion of many, fully authorize its adoption in future. Few, I am sure, hold that excellent surgeon in higher estimation than I do, yet I cannot withhold expressing what I fully believe experience will justify me in stating. It is universally acknowledged amongst writers, that a woman is unsafe until the placenta is expelled, and who has not witnessed the uneasiness both of patient and friends until this is accomplished, proving a general consent to the same fact? In case No. 5, I left it, and the consequence was that it was detained two months, during which time the patient was never well; and at length it was expelled with a fearful hæmorrhage. In the sixth case it was left, and for six days both medical attendants, patient, and friends were in constant doubt of something going wrong, but to our relief it was then expelled, a disgusting mass. These, however, I consider good escapes, rather than proofs of good treatment, because I find that reports of more serious consequences abound in the writings of those who have enjoyed the widest fields for observation. Dr. BURNS says, p. 297,

'If the gestation has not advanced far, the placenta may be expelled in small portions for many months, but the patient is all the while languid, hysterical, and subject to irregularities of the menstrua, and very often to obstruction. But more frequently the symptoms are very acute. We have loss of appetite, prostration of strength, tumid or tender belly, frequent small pulse, hot skin, and various hysterical symptoms. The discharge from the vagina is abominably fetid, and hæmorrhage sometimes occurs to a violent degree.'

Mr. INGLEBY's observations are to the same purport, and he relates a fatal case from the placenta being retained. (p. 118.)

SMELLIE gives a case from Mauriceau, vol. 2, p. 171, strongly proving the same point.

A woman miscarried at the fourth month, and the placenta was left entire. The midwife said the womb had closed, and she could not extract it. He saw her on the third day, and found her statement correct. He could not remove it. She was at the brink of the grave, from 'a prodigious flooding.' The placenta became putrid, and a most violent fever ensued. 'accompanied with exacerbations two or three times a day, and with faintings and other symptoms usual on such occasions.' The placenta was at length expelled, and the woman recovered.

Smellie mentions another example, which happened in the practice of one of his pupils. (Vol. 2, p. 167.)

The placenta was retained after an abortion at the fifth month. The patient was subject to constant draining for three months, until she became pale and emaciated. At this time he visited her. The os internum admitted two fingers, with which he was enabled to separate a hard round substance from its attachment to the uterus, but could not bring it away. He then used a blunt hook, and removed it in three portions. The discharge ceased, the woman recovered, and afterwards bore children.

The above cases clearly prove the great importance, and, in my humble opinion, the absolute necessity, of removing, in all possible cases, the whole of the uterine contents; and here I must especially urge the removal being accomplished as quickly as possible. It is a remarkable, but a well-established fact, that the uterus and its orifice become almost permanently contracted very shortly after even a partial expulsion of its contents. Cases No. 1 and 7 in this paper show the strong tendency to this contraction, but other cases prove it completely. MAURICEAU mentions a person who miscarried at the sixth month; part of the after-birth remained, and before he arrived the os uteri was so closed he could not effect its removal. Dr. DEWEES dwells particularly upon this fact. (P. 414.)

I feel it, however, a duty to state, that Dr. DENMAN, who appears to be the oracle of British accoucheurs, and in many respects most deservedly so, does not advocate the removal of the placenta when retained after abortion. His concluding remark is as follows. (4to. ed. p. 490.)

'At all events, much less mischief may be expected from the retention of a putrid placenta at this time, than from attempts to force it away by the medicines usually given for that purpose, or by manual assistance.'

I would simply beg to make this observation. I can find no argument to justify the use of 'force' in this case, but between the mischief arising from a judicious endeavour to remove a retained placenta, and that arising from a putrefying substance within the Uterus, an organ possessing sympathies so numerous and so important, I leave the reader to decide."

The cases themselves we are unable to insert, and they cannot be abbreviated.

### CREPITATIO MUSCULORUM—A NEW, OR VERY RARE AFFECTION.

We have recently seen a very remarkable instance of this curious complaint. A lady of distinction and title, aged between 60 and 70, had enjoyed the most perfect health till within these few years. About that period she experienced some anomalous feelings about the head, indicative of fulness of the cerebral vessels, and these were accompanied and followed by certain vitiations of the senses of tasting and smelling. These symptoms she disregarded, her health being, in other respects, pretty good. Gradually, however, she began to perceive a weakness in the left lower extremity, with some uneasy sensations in the line of the sciatic nerve between the hip and the knee. This debility steadily increased, during the last twelve months, and she is now obliged to go on crutches. The muscles of the thigh, but not of the leg, are flabby and a little wasted. She can move the extremity in every direction; but has not the power of leaning in the slightest degree upon it. The most singular phenomenon, however, is this;—that, whenever she either bends or extends the knee, a crackling noise is distinctly heard, even at a considerable distance, along the course of the rectus femoris muscle, exactly resembling the cracking of fingers when stretched. The action of flexion and extension is accompanied by painful sensations in the rectus femoris, the two vasti, and the cruræus. When at rest, she feels no pain. Mr. Brodie and the senior Editor of this Journal examined the thigh with the greatest care. The ear was repeatedly applied along the muscles above-mentioned, and the crackling noise was loudly heard. As the ear approached the knee-joint, the noise diminished, and it was perfectly evident that the sound did not proceed from the joint, but from the muscles of the thigh. In the whole course of his experience, Mr. Brodie never met with but one case at all resembling this. It was a gentleman who complained to him of such strange and anomalous sensations about the right shoulder and side of the back, that he thought for some time the gentleman was hypochondriacal, and fancied a host of morbid and unintelligible feelings. In one of his visits the patient asked Mr. Brodie what could be the cause of the cracking noise in his back. Mr. Brodie had not been made acquainted previously with this symptom. But the patient soon convinced him of its reality; for, on moving the arm up and down, there was a cracking noise emitted from the muscles about the shoulder, and especially the latissimus dorsi, so loud that it could be plainly heard even in an adjoining room. Mr. B. examined with the ear, and clearly ascertained that the noise proceeded from the muscles, and not from the shoulder-joint. In this case there was no diminution of muscular power, but only painful sensations. The gentleman, in the course of some years, got rather better; but Mr. B. lost sight of him, and the final result is not known. In respect to the lady, various means had been used, and half a score of physicians and surgeons had been consulted, without the slightest benefit. She was finally recommended to go on a tour up the Rhine in August of the present year, more to divert her mind, and give a fillip to the general health, than with any prospect of recovery from the local complaint. She had heard so much of the "Bubbles of the Brunnens," that she was anxious to try their efficacy. They will probably burst and leave no mark of their power behind.

What is the nature or the cause of the phenomena abovementioned? We believe it would puzzle a Philadelphia lawyer to solve the problem. For our own parts, we are completely *pozed*—and we shall not trouble our readers with any of the hypotheses we have formed to unriddle the mystery. The lady, who is very intelligent, says that she feels as if the muscles were dry, and snapped or cracked, with some pain, when she puts them in motion. By the way, can any one explain the cracking of the finger-joints when forcibly extended? It rarely happens that a joint can be made to crack more than once, however often the extension may be repeated at that time. Is it the forcible separation of articulating surfaces that were in perfect contiguity? All separations of such surfaces produce a noise. But why can it not be repeated till after a period of time? We shall keep an eye to the case above-mentioned, and communicate the result to our readers.

---

### Hydatids of the Kidneys passed by the Urethra.*

Elijah Jones, æt. 27, a comb-maker, of pale complexion and slender form, applied to Dr. Duncan on the 13th May. He brought with him several portions of a membranous-looking substance, having a pearly, semi-opaque, pulpy appearance, and which he said he had passed with his urine three days previously. He stated that he made water rather oftener than usual, and sometimes with difficulty; and that he had a constant shooting pain in the perinœum, which was increased after micturition. He had also occasionally a sense of 'weakness' in the right lumbar region. Urine of natural appearance; and functions natural.

On examining the substances above-mentioned, one was discovered of a globular shape, and about 1¼ inch in circumference,—evidently an Hydatid, of the genus Acephalocyst. It was filled with a transparent fluid, having floating in it another very small hydatid, which gravitated in the surrounding fluid. The remainder of the substances consisted of the coats of seven or eight hydatids which had burst, and which, when filled with water, varied in bulk from the size of a pea to that of a pigeon's egg.

He stated that, seven months ago, he got a "bad cold," and suffered from pain above the right hip, and in the perinœum; and that five months ago, a blister was applied, which removed the pain above the ilium, but that he still feels occasional uneasiness there. About a month ago, he passed several hydatids, which caused some obstruction to the flow of urine, but no more appeared until three days ago, although during the last month he has had constant pain in the perinœum, apparently near the neck of the bladder.

He was ordered to take *diluted Muriatic Acid,* 12 minims three times a day.

16th. Another hydatid has been passed (burst). The pain is nearer the end of the penis.

24th and 25th. Two more hydatids passed, which obstructed the urine for some time. No pain in the perinœum now; it is generally felt six or seven hours before the hydatid is expelled.

June 3d. No more hydatids have appeared. Complains only of weakness in the back and hip.

The above case is interesting from the extreme rarity of its occurrence. Dr. Craigie says, "that the uterus is the only cavity, with mucous surface, in which inspection shews that hydatids have been found:" and there can be no doubt that, in this case, they were formed in the kidneys, and probably increased in size after their descent into the bladder.

The following account of the *post-mortem appearances* in one of the few instances of the kind on record, is taken from the *Philosophical Transactions* for 1687. Dr. Tyson, in stating what was observed in the bladder, says, "therein, upon Apertion, we discovered a very strange sort of Cystes or Bags, of the exact Figure of Eggs, of several dimensions, some larger than Goose Eggs, others as big as Hen Eggs, to the number of twelve in all; and about eight of them whole and repleat with a limpid Serum;.... all of them loose and free, without the least adhæsion, either to one another or to the Coat of the Bladder.... nor could we imagine that this miserable patient could possibly make any Water but what happened upon the breach of some of these watery Tumours, when the Bladder was crouded beyond its dimensions..... The *Vreters* were of the largeness of the Small Gutts in Children, so that they could easily admit two fingers into their

---

* Liverpool Medical Journal, July, 1834.

Cavity..... One of the *Vesiculæ* being opened, had a large cluster of small *Ova* as big as Grapes, all repleat with Liquor. All the rest contained nothing but *Serum.*" Two small ova were observed at the entrance of each Ureter, having descended from the kidneys.

---

### OBSERVATIONS ON CATARRHAL AND CATARRHO-RHEUMATIC OPHTHALMIA. *By* FRED. TYRRELL, *Esq.*

In our quarterly contemporary for July last, there is a good practical paper on the above subject, from the pen of a good practical surgeon. Mr. Tyrrell remarks that, during the concluding part of last Winter, and up to July of the present year, catarrhal ophthalmia has been more prevalent than he ever knew it to be. The disease was often so severe as to affect the sclerotic as well as the conjunctiva. The following is Mr. Tyrrell's description of the—

### CATARRHAL OPHTHALMIA.

"It commences with stiffness and heaviness about the palpebræ, with slight irritation or itching of the ciliary margins, and a sensation as if some fine particles of dust had lodged on the surface of the globe; there is usually slight intolerance of light, with increased secretion of tears and agglutination of the lips during sleep. After a short time the patient experiences pain, which is increased by motion of the eyelids, or by exposure to bright light. These symptoms are aggravated about sunset.

On examining the eye, there is perceived in the early stage slight thickening and redness at the free margins of the lids, and probably some coagulated secretion collected at the inner canthus and about the cilia. The ocular portion of the conjunctiva exhibits a few tortuous vessels filled with red blood, whilst the palpebral portion is red, tumid and villous, like crimson velvet; and in the lower folds between the ocular and palpebral parts of the membrane a little whitish or slightly yellow and thick mucous secretion is lodged.

The inflammation, however, soon spreads to the ocular conjunctiva, the vessels of which become extensively injected with red blood, and, on close inspection, are found to form a beautiful net-work. In severe cases, the conjunctiva is partially thickened and elevated by deposition of serum between it and the sclerotic, constituting a partial chemosis, and occasionally small spots of ecchymosis are perceived from the rupture of some minute vessels.

If the disease extends farther, it produces ulceration of the cornea.

This ophthalmic disease seldom exists without some symptoms of general catarrh, such as headach, sense of fulness about the frontal sinuses, sneezing, increased secretion from the nose, and some general febrile symptoms: the local and general diseases are usually reciprocal.

In young subjects the catarrhal-ophthalmia is often modified by a strumous diathesis, which occasions the local affection to be more severe, augments especially the intolerance of light and lachrymation, and disposes to the formation of pustules.

This disease may occur at any period of life. It is produced by a peculiar state of the atmosphere, during the continuance of which there is considerable difficulty in entirely removing the ophthalmia."*

The treatment must be modified by the severity of the symptoms, the extent of the constitutional disturbance, and the condition of the general power.

"When there is simply affection of the palpebral conjunctiva with morbid mucous secretion and little general disturbance, and sufficient constitutional power, I prescribe a brisk aperient of calomel and colocynth, or calomel and rhubarb, or jalap, and afterwards a small quantity of some of the preparations of mercury with antimony, or with Dover's powder at night, and a mild saline aperient in the morning, also mild diet, principally vegetable and fa-

* MED. QUART. REV. No. IV. p. 416.

rinaceous, with quiet, and the avoidance of cold or damp, or of bright light.

Locally, I employ merely tepid water, to cleanse the eyes; or warm water to foment them when uneasy, and some simple ointment, which should be smeared upon the lids and eyelashes before the patient goes to sleep.

When the local disease is acute and the constitutional affection considerable, general or local bloodletting must be resorted to; the former being only necessary in persons of naturally full habit or subject to inflammatory diseases; and after bloodletting the treatment above described will be applicable.

In a few days the mild or acute form, if treated as I have described, passes into the chronic stage, which is denoted by a general mitigation of symptoms, but more especially by an alteration in the mucous secretion, which becomes thin and pale, and by the change in colour of the conjunctiva, which loses its brilliant aspect, and presents a more pale and lax appearance; at the same time it is found that the general power is failing under the continuance of the constitutional affection; the treatment must therefore be changed. Instead of exciting the secretions, they must now be kept as near as possible in their natural state, and a more generous diet must be allowed, but still the patient must abstain from such food as may tend to excite vascular action. It is necessary, in persons of feeble power, to give, in addition, some medicinal stimulus, as some of the preparations of bark, mineral acid, or ammonia.

To subdue the remains of the local disease, a slightly astringent lotion must be substituted for the tepid water, as a solution of the acetate of lead, half a grain, or a grain to the ounce; or a solution of alum, a grain or two grains to the ounce; the sulphate or acetate of zinc, a quarter or half a grain to the ounce, or nitrate of silver in the same proportion. Also a stimulating ointment must be applied instead of the milder form, as the dilute citron ointment composed of half a scruple or a scruple of the unguentum hydrargyri nitratis, to two drachms of unguentum cetacei, or a scruple of the unguentum hydrargyri nitrico-oxydi to a drachm of the unguentum cetacei."

Where a strumous diathesis prevails, and where, as was observed, there is much intolerantia lucis, counter-irritation by blisters is necessary. The intolerance exists both in the acute and chronic form, which renders it difficult to distinguish between them, though it is necessary to discriminate in the treatment. Mr. T. is convinced that more benefit results from repeated blisters, than from keeping one open. Cold applications are injurious to the eyes of strumous children. He uses tepid water in the acute stage, and trusts to general remedies in the chronic. We now proceed to the—

### Catarrho-Rheumatic Ophthalmia.

"This affection commences with symptoms similar to those of the catarrhal, but with more early suffering: for in addition to the symptoms of the catarrhal disease, the patient experiences a constant dull aching pain in the globe of the eye, and in the temple and eyebrow: the globe feels tense, and as if it had been bruised; and these pains and sensations become so much augmented towards evening as to prevent sleep. The accession of pain is accompanied with intolerance of light and a feeling of dryness of the conjunctival surface, so that the motions of the eyelid upon the eye produce excessive pain; but occasional gushes of tears occur, which for a few moments produce some relief; while the symptoms continue thus acute, the globe of the eye, the temple, and eyebrow, are extremely tender to the touch.

In the intervals between the severe attacks, when there is but slight intolerance of light, vision is frequently indistinct, and occasionally small black spots or muscæ are perceived by the patient.

On superficial inspection of the eye, the appearances denoting catarrhal ophthalmia of an acute kind are apparent; such as the red palpebral margin, the thick mucous secretion, the thickened and villous state of the palpebral conjunctiva, with the tortuous vessels and vascular net-work on the ocular portion of the membrane; but an attentive examination soon discovers that the colour of the ocular conjunctiva is more uniform and deeper than in the simple catarrhal disease: and this upon further observation is found to depend upon the existence of another set of minute vessels filled with red blood, which can be traced in the intervals between the tortuous vessels of the conjunctiva as passing beneath them in a straight course from the margin of the cornea towards the orbit; these vessels are situated in the sclerotic coat; they are occasionally found more abundant in one part than another; as, for example, more upon the nasal than the temporal side of the cornea, and of course augmenting the depth of colour in such particular situations: it requires therefore a careful examination of the whole ocular surface to detect them.

When the vision is affected, being cloudy or troubled with muscæ, some change is perceptible in the iris; its pupillary aperture is contracted, its brilliancy is diminished, or its colour slightly altered; which circumstances are best detected by comparing the iris of the affected eye with that of the healthy organ: the motions of the affected iris also are sluggish. The iris suffers in consequence of inflammation extending to it from the sclerotic.

Sometimes a partial or complete ash-coloured line is seen between the cornea and vessels injected with red blood, most frequently the line forms a complete circle around the cornea; but I have seen it merely occupying a portion of the circumference of the cornea, being apparent at its temporal and nasal margins, and deficient superiorly and inferiorly. This appearance is considered by the continental and also many of our own ophthalmic surgeons, as a diagnostic of rheumatic inflammation. Such opinion, however, I consider to be erroneous, for I have seen a similar line present in iritis, simple sclerotitis, and choroiditis. I believe it results from the mode of junction between the sclerotic and cornea, occurring when any of the diseases alluded to are present, and seen most distinctly when the junction of the two structures is very oblique, but not at all evident when the textures are joined with little or no obliquity. Now and then the margin of sclerotic overlaps or joins obliquely the circumference of the cornea, more in one part than another, and this I have observed most frequently on the temporal and nasal sides, so as to make the transverse diameter of the cornea less than the perpendicular diameter. When such formation exists and inflammation affecting the sclerotic occurs, the ash-coloured line is apparent only towards the nose and temple."

The exciting causes are cold and moisture; and Mr. T. suspects that the simple catarrhal affection is often converted into the rheumatic, by the injudicious application of cold lotions.

### Treatment.

When inflammation preponderates in the conjunctiva, and the sclerotic is but slightly affected, leeches must be applied, and brisk purgatives given. For a few days, Mr. T. employs the same means as in the simple catarrhal affection, excepting that, when the circum-orbitar pains are severe, he directs blisters, or a few grains of mercurial ointment, with opium, to be rubbed on the temple and forehead at night. As soon as the severity of the conjunctival affection has subsided, he prescribes a more nutritious diet, and small doses of cinchona and soda, sarsaparilla and lime-water, or some mild tonic with an alkali. Locally, warm water is occasionally used to cleanse the eye; "but the patient must carefully wipe it, and not allow the surface of the lids to remain moist, as I am confident it augments the mischief." The repetition of blisters is serviceable where there is much intolerantia lucis, or continual circumorbitar pains. When irritability, with little vascularity, remains, a drop or two of vinum opii once or twice in the 24 hours, dropped into the eye, is useful.

"If the catarrhal affection of the conjunctiva be slight, and the inflammation of the sclerotic severe, without, however, much affection of the iris, local bloodletting is rarely necessary. The patient is at first to be freely purged by some drastic medicine, after which a full dose of colchicum, with a narcotic, may be given, especially when there is any marked rheumatic diathesis: the best period for administering this dose is in the evening, a short time before the usual period of exacerbation. I have known it to subdue altogether the sclerotic inflammation, leaving but a slight catarrhal ophthalmia. If the first dose appears to alleviate the disease, it may be repeated every six or eight hours for three or four times; but I have never found any benefit from a longer continuance of this remedy, nor have I seen much advantage from it when it has occasioned disturbance of the stomach or bowels. I consider its beneficial action very uncertain.

Otherwise, I prescribe according to the state of general power, which it is of the utmost importance to observe, in order to treat successfully. Very often, when the local disease appears acute, the general power is extremely feeble; or, on the contrary, when the local disease is slight, the constitutional power is good; but occasionally, the general action is energetic when the local disease is severe. When the action of the heart and arteries evinces deficient power, although the local symptoms and appearances may indicate an acute form of disease, any active depletion likely to affect the system ought not to be adopted; blistering is the best means of relieving the local congestion; besides which, medicines should be given to correct any error in the principal secretions, especially those of the bowels, skin, and kidneys: the two latter I find most frequently to be deranged. Antimonials with salines, are therefore most serviceable after the bowels have been acted upon. The patient must be allowed a good nutritious diet, and even a small quantity of stimulus, if much addicted previously to its use.

As soon as the secretions are in a healthy condition, tonics should be exhibited, and if the general power is good I prescribe five grains of the pulvis cinchonæ, and an equal quantity of the sodæ subcarbonas exsiccata every four or six hours; and I know of no remedy so generally serviceable in the state of the disease above described. I am indebted to Mr. Wardrop for the suggestion of this medicine. I have frequently known it succeed in relieving inflammation of the sclerotic, when the larger doses of the same preparation have been taken some time without benefit. Should this remedy fail, I give the sarsaparilla with lime water, but with either medicine it is necessary to administer an occasional purgative, with or without some mercurial, according to the state of the biliary secretions.

When the patient's power is feeble, I usually commence with the sulphate of quinine, as a tonic, in doses of three or four grains every four or six hours; and if there is a disposition to inordinate cutaneous secretion, I prescribe, in addition, twenty or thirty minims of the dilute sulphuric acid. Under these circumstances, also, a generous diet is requisite, and a moderate quantity of any stimulus to which the patient may have been accustomed. I have lately seen many cases in which the catarrho-rheumatic disease had continued (principally however of a rheumatic character) for six or seven weeks, or more; a depletory treatment having been persisted in, and in which a cure has been effected in as many days after the tonic plan has been adopted.

It sometimes happens that the disease has extended from the sclerotic to the iris before the patient applies for relief, or that the affection being treated under the supposition that it is merely a conjunctival inflammation, the remedies employed have not been sufficient to prevent the extension of the disease to the iris. Inflammation of the iris is indicated by a loss of brilliancy, an alteration in colour, a thickening of the pupillary margin, and a contracted and sometimes irregular condition of the pupillary aperture. When the iris is but slightly diseased, there is no occasion to deviate from the treatment which I have just described; but if the inflammation of this texture is decidedly developed, it is absolutely necessary to resort to mercurial treatment to stop its progress, otherwise the plan of depletion which I direct in the early stage of catarrhal or simple catarrho-rheumatic ophthalmia, may not arrest the inflammatory action in time to prevent deposit of lymph in the texture of the iris and in its pupillary margin, by which the pupil may be rendered permanently irregular, or its margin become in part adherent to the anterior capsule of the lens, and the transparency of the capsule itself be materially injured.

The exhibition of mercury must be carefully and guardedly resorted to, especially when the constitution is feeble, which I have observed it most frequently to be in persons suffering from this disease; nevertheless I deem the use of the remedy of the greatest importance, if given in small doses, at short intervals, whilst the patient is allowed a nutritious diet, and the secretions kept in good order; the surgeon may, by keeping a careful watch, always stop the exhibition of the medicine in time to prevent any mischief.

At the same time that mercury is given, the extract of belladonna should be applied to the eyebrow night and morning, to keep the pupil in a dilated state, and prevent any adhesions taking place between the pupillary margin of the iris and the anterior capsule of the lens, with a contracted pupil; or to aid in the separation of such adhesions, should they have been formed previously.

The mercurial treatment must be stopped as soon as the inflammation of the iris subsides, whether the system be affected by the remedy or not. I have seldom had occasion to give mercury so as to produce any marked effect on the system in these cases, and have always found that when the remedy has by accident, or inattention, created much general disturbance, the cure of the remaining disease in the sclerotic has been more difficult. The disease in the iris being subdued, the plan I have recommended for relieving the inflammation of the sclerotic should be immediately adopted.

In several instances which have come under my observation during the last few months, although the general and local diseases have not been very severe, the patients have experienced excessive debility, and have not recovered until they have had the benefit of change of air for a short time.

I have been desirous to shew, in the preceding observations, that it is of much importance, by careful examination of the organ and attention to symptoms, to ascertain the extent of disease before treatment be commenced, and that the treatment must be varied according to the extent and severity of the inflammation; but especially to point out the existence of apparently severe local inflammation, with a feeble state of the constitution, which I have observed frequently during the late prevalence of these diseases; for I am satisfied that inattention to this circumstance has been the principal cause of want of success in the management of most of the cases which I have seen in consultation."

We have thus laid the whole substance of Mr. Tyrrell's paper before our readers, as it will prove serviceable to the practitioner in the daily rounds of his avocation.

---

RESEARCHES ON THE DIAGNOSIS AND PATHOLOGY OF ANEURISM. By Dr. W. STOKES.

[Dublin Journal, July, 1834.]

This is a long and valuable paper, and relates, of course, to internal aneurisms; it is, therefore, in the domains of the physician. We shall, in imitation of the author, proceed at once to the cases.

CASE 1. *Aneurism of the Hepatic Artery—Jaundice—Death.*

S. Mears, aged 35, of regular habits, but who had had an attack of apoplexy, was admitted into the Meath Hopsital, 7th August, 1832, in a state of complete jaundice. Nine weeks previously he was attacked with copious hæmatemesis of five days duration. The yellowness commenced nine days before he entered the hospital. He had yellow vision, with nausea, thirst, and some epigastric pain, with pulse 112, and the fæces and urine as in jaundice. The left lobe of the liver could be felt much enlarged, and also the right, whose edge stretched down to the umbilicus. Two inches to the right of the umbilicus was felt a soft pyriform and fluctuating tumor, considered to be the gall-bladder distended. In this state he continued for nine days, when he became covered with a miliary and afterwards a petechial eruption. In none of the examinations did they detect any pulsation in the tumor. On the 17th of August he was sitting up in bed, when he fainted, and suddenly expired.

*Post-mortem Examination.* On opening the abdomen a layer of recently coagulated blood covered the surface of the intestines. The liver was found to be small, and beneath its thin edge were seen two projecting tumors—one, the gall-bladder enormously distended —the other occupying the notch in the anterior edge of the liver, the size of an orange, roughly coated with cellular membrane, and without fluctuation. The liver had been pushed from behind forward by these tumors, giving it the enlarged appearance in the living body. The aorta was carefully examined, and found to have no communication with the tumor. A rigid investigation was then made by several medical gentlemen, and the tumor proved to be an aneurism of the hepatic artery covered by the capsule of Glisson, and so situated as to pass directly under the bile ducts. The opening of the vessel was a well-defined slit, and seemed to be the result of a perfectly local disease. The state of the biliary ducts throughout the liver, being enormously dilated up to the point of obstruction. The larger ducts would admit the point of a man's thumb. The dilatation continued to the peritoneal surface of the liver, forming numerous projections, varying from the size of a wallnut to that of a pin's head, apparently by the distention of their ultimate ramifications. They contained bile, which spirted out with force when they were opened. The substance of the liver was soft and friable. No disease in any part of the gastro-intestinal canal.

Dr. Stokes has made some ingenious observations on the above very curious case, especially as respects the diagnosis. We much doubt whether any of the phenomena recorded in this instance will enable us to ascertain an aneurism of the hepatic artery in the living body; but the case itself is important, as shewing how easily we may be deceived as to enlargements of the liver, by the existence of tumors in its vicinity. There is one observation in these remarks which struck us forcibly, and not without some sense of humiliation. "I feel little doubt (says Dr. Stokes) that the violent impulse of the heart in phthisis is, to a certain extent, produced by the circumstance, that the lung has lost much of its elasticity, and thus offers greater resistance to the motions of the heart." For the edification of the young pathologist, we candidly confess that we have many times pronounced disease of the heart, when, on dissection, the heart was sound, and the neighbouring lung was the seat of disease. It is more difficult to discriminate between increased action and increased structure in the central organ of the circulation, than most pathologists imagine. The longer we have lived, the more convinced are we of this difficulty; and scarcely a day passes without offering us examples where we are at a loss to say which of the two states is the cause of the phenomena presented to the senses by auscultation. Let this confession from a senior in observation, moderate the pride and increase the assiduity of the junior pathologist!

Case 2. *Aneurism of the Arteria Innominata.*

This was a shoemaker, admitted into the Meath Hospital, 29th Dec. 1833, being 34 years of age, stout and muscular. He complained of cough, dyspnœa, pain in the chest and head. Next night he was seized with hemiplegia of the left side. The symptoms abovementioned came on five weeks previously, after exposure to cold. On examination the chest sounded well over its whole extent, excepting at the sternal extremity of the right clavicle, where it sounded dull. The respiration was intensely puerile in the left lung—exceedingly feeble in the right, but without râle. On applying the stethoscope to the sternal extremity of the right clavicle, a very loud double pulsation was heard, with strong impulse, diminishing in intensity as the heart was approached, the sounds and impulse of which were natural. On pressing the fingers behind the clavicle, a small pulsating tumor could be felt in the direction of the arteria innominata. No *bruit de soufflet* could be detected in any part of the chest.

The symptoms were materially relieved by local bleeding, cold applications, purgatives, &c. but on the 5th January last, the symptoms became all aggravated, and he was attacked with diffuse cellular inflammation of the integuments of the neck, extending from the clavicle to the inferior maxilla. The swelling was soft and elastic, very tender, and with distinct crepitus. The face was livid and swollen, and the veins of the head and neck much gorged—the breathing was laborious, and there was loud tracheal râle. By various and judicious means the symptoms were alleviated from time to time; but he ultimately sunk on the 17th February, 1834, after the physical lesions had become multiplied and extensive. The tumor above the clavicle had attained the size of a hen's egg—dullness of sound extended over a great part of the anterior of the right side of the chest—pectoriloquism was decided in the tumor—and there was intense bronchitis in the left lung—breathing stridulous — voice often inaudible — headache, which was a constant symptom.

*Dissection.* Much fluid between the membranes of the brain, and an abscess in the right hemisphere, containing an ounce of matter. The right lung was collapsed, and reduced to a dark carnified mass. Strong adhesions, and much fluid between the pleuræ. The left lung was very voluminous, and its tubes filled with mucus. Two ounces of serum in the pericardium. The aorta was diseased, being somewhat dilated, and its coats thickened, the inner one being nodulated, and speckled with yellow spots. The aneurism was of the arteria innominata, the whole front of which artery was destroyed, and replaced by the wall of the sac. The posterior half was sound. The aneurism rose at first narrow, but gradually increased in bulk till it equalled a large cocoa-nut, the walls being of irregular thickness. It was filled with large fibrous and laminated coagula. By its pressure it had so flattened the right side of the trachea, that the free margins of the cartilages overlapped those of the opposite side, and almost annihilated the calibre of the tube. The right carotid and jugular vein, on the posterior surface of the sac were flattened and obliterated. The vagus nerve was also flattened, and its fibres were vascular. The right and left venæ innominatæ were flattened and completely obstructed.

Dr. Stokes gives an admirable exposition of the symptoms during life, as explained by the *post-mortem* examination. We regret that our limits prevent us from giving more than one passage which, however, conveys a very valuable hint to the pathologist, and especially to the zealous auscultator.

"Let us now consider the symptoms as connected with the chest. One of the most remarkable of these, was the difference of the intensity of respiration in the two lungs, the sound of percussion of which was every where clear, except in the immediate situation of the aneurism. This circumstance, arising from the compression of one bronchus, seems to me a most important one in the diagnosis of thoracic aneurisms, and one to which sufficient attention has not been paid. In the case to which I before alluded, published by Mr. Porter, this phenomenon was well marked, and was in truth the only unequivocal sign of any tumor existing in the chest; and there is at present a patient in the hospital labouring under many symptoms of aneurism of the aorta, though no external tumor is perceptible and who presents this sign in a most remarkable manner. When we consider the difficulty that has hitherto been attendant on the diagnosis of thoracic aneurisms, *previous to the appearance of tumour*, any additional sign must be considered as of great value. I have no doubt that this one will in many cases prove highly valuable. The observation, too, may to a certain degree apply to the diagnosis between chronic laryngitis and the pressure of tumours external to the tube; for if, previous to the occurrence of the stridulous breathing, this difference of respiration has been observed in the lungs, a difference inexplicable by any result of percussion or auscultation of the respiratory murmur, it will give almost a certainty that the symptoms are not the result of original laryngeal or tracheal disease, but of gradually increasing pressure, first upon one bronchus, and afterwards on the trachea itself."

We fear that the diagnosis of thoracic aneurisms will ever remain obscure, on account of the various directions which the tumor may take, and the different sensibilities of the parts incommoded. The foregoing case, however, which we recommend in the original to every pathologist, exhibits a striking example of the accuracy of diagnosis by the stethoscope, in respect to the lungs, pleura, &c.

Case 3.—*Dilatation of the ascending Aorta—Death by Rupture.*

A man, aged 30, was admitted Jan. 18th, 1831. In the preceding September, he had had a fall on his right side, succeeded by pain in that situation. A month afterwards, he became affected with a sharp pain in the upper part of the *left* side, which continued for some weeks, when he perceived a pulsation between the cartilages of the second and third ribs, from which time the thoracic pain diminished. A flattish tumor was now felt extending from the second to below the third rib, presenting a double pulsation, closely resembling those produced by the action of an excised heart, and without any *bruit de soufflet or rape*. He had no dyspnœa in the erect position; but difficult breathing the moment he lay down. The stethoscope detected nothing unnatural in the heart or lungs. *Low diet, bleeding, digitalis.* He improved till the latter end of February, when he was attacked with severe pain in the chest, numbness of the left arm, and inability to lie on the right side. Some relief was obtained by local bleeding and by blistering; but he was unable to use the recumbent posture. The double pulsation could be plainly felt. A bleeding from the arm relieved the pain, and enabled him to lie down. He continued in this state till the 11th of April, when he suddenly expired.

On dissection, the ascending aorta was found to be greatly dilated, the dilatation commencing at the semilunar valves, and terminating at the origin of the innominata. The tumor adhered to the left side of the sternum, and to the cartilages of the second and third ribs. It was about the size of a goose-egg. It had ruptured into the pericardium, which was found distended with coagulated blood and serum. The heart itself was healthy, and the lungs crepitating.

Case 4. *Aneurism of the Thoracic Aorta, without external Tumour, recognized during life.*

P. Walsh, aged 26, was admitted 23d July, 1832, complaining of cough and dyspnœa, to which he had been subject for nearly two years, but much aggravated within ten days. His neck was generally enlarged as if a tippet had been put round it; the jugular veins being turgid and tortuous. He had short cough, and mucous expectoration, with stinging pain in the right shoulder —pulse 100—impulse of the heart slightly increased, and its sounds heard over a large portion of the chest. On the left side some *bruit de rape* accompanied the first sound of the chambers. The anterior portions of the chest were less sonorous than natural. In the right subclavicular region, the respiration was feeble, with a slight mucous râle—in the left lung, the respiration was puerile. "On laying one hand over the right scapula, and the other under the clavicle, a distant though distinct impulse could be felt, apparently synchronous with that of the radial artery." The patient had some dysphagia.

Connecting the foregoing phenomena with the history of the case and the auscultic indications, Dr. S. came to the conclusion that, in all probability, there was aneurism of the arch of the aorta. He was treated by small bleedings, local and general, and was so much benefited that he left the hospital. Soon afterwards he got ill, and went into another hospital, where he died suddenly; and, on dissection, an aneurism of the arch of the aorta was discovered. It had burst into the pleura.

Dr. Stokes has appended some good remarks on the subject of internal aneurisms generally, and the signs by which they may sometimes be recognised, for which we must refer to the paper itself. Dr. Frazer has also communicated two or three cases of aneurism from his military experience, of which we shall not be able to give a satisfactory account, in consequence of our closing limits.

The first case was that of a British soldier, who came into hospital complaining of violent pain in his back, particularly along the spine, but without any morbid signs of local or constitutional illness. His health appeared good, and there was no emaciation. Some trifling remedies were prescribed, and it was suspected that he was feigning. He left the hospital, but again returned, and again the medical attendants were puzzled and suspicious.—They were not a little alarmed and mortified, however, on learning that the poor fellow dropped down dead, a few hours after he came into the hospital. On examination, a large aneurism of the abdominal aorta was found ruptured, the bodies of the adjoining vertebræ being carious.

The second case was that of a gentleman, aged 30, who was attacked in the latter end of 1830, with derangement of bowels, followed by the feeling of a tumour in the left hypochondrium extending to the groin, with weakness and pain in the back. These symptoms increased, till at length a pulsating tumour appeared in the postero-inferior portion of the left side, which rapidly increased in size. As the case was considered to be aneurismal, Valsalva's system was adopted. His sufferings were increased so intolerably that he wished for death. On one occasion, in a fit of despair, he entered a tavern, ordered a sumptuous dinner, and drank a pint of wine, which was followed by a cessation of his distressing symptoms for several days. He always found that low living increased his sufferings. Dr. Frazer was unable to detect any tumor in the side. He became affected with atrocious colics—his legs swelled—and he died. On examination, a large fluctuating tumor was found extending from below the left scapula to the last rib. The left pleura was filled with coagulated blood, and its separated serum. This had compressed the lung, and displaced the heart to the right side of the chest. In the lower portion of the pleural cavity was perceived the ruptured aneurismal sac, of great size, displaying its concentric layers of coagulable lymph, hanging in irregular masses in the pleural cavity. Five of the vertebræ were deeply eroded. The left crus of the diaphragm was destroyed, and the muscle partly enveloped the tumor in the form of an arch. The heart was flabby, shrivelled, and of a livid hue. Near the bifurcation of the aorta was a well-defined oval perforation of the posterior surface, communicating with the sac.

We have now condensed the most important imformation contained in this long and interesting paper of Dr. Stokes, and beg to offer him our thanks for the benefit which we have received from its perusal.

---

A Practical Treatise on Medical Jurisprudence, &c. &c. By J. Chitty, Esq. Barrister at Law. Part the First. Royal octavo, pp. 466. July, 1834. Price One Guinea, with Plates.

The Doctor is situated very differently from the Lawyer in a court of justice, and when a subject of medical jurisprudence is under investigation. The medical man is entirely passive—in fact, he is a witness brought forward to elucidate points of medicine (including all its branches of course) *alone* and without any reference to the law of the case. The lawyer, on the other hand, questions the witness on a subject with which he is not supposed to be conversant, and consequently the more he knows of medicine, the better qualified he is, not only to elicit truth, but to puzzle the doctor for the sake of his client. Thus, if a medical man was perfect in all branches of his science, and never read one word of law, he would give as good evidence as if he had all the legal knowledge of Blackstone, Coke, or Littleton. This shews how much more important it is for the counsellor to study physic, than for the physician to study law. Is the physician ever examined on the law of the case in any court? We never saw such an instance. To the medical reader Mr. Chitty's work may appear to have entered a great deal too far into medical matters, and, in fact, to form a *popular* digest of medical science, for which *they* have no occasion. But Mr. Chitty clearly saw that the medical part was wanted by non-professional and not medical people, as his title-page renders evident; for it states that the work is designed for "members of Parliament, lawyers, coroners, magistrates, officers in the army and navy, and private gentlemen." Not a word is said about medical practitioners among the anticipated readers; but he adds that the work *will* contain, "all the laws relating to medical practitioners." In his preface he distinctly informs us that the *fourth* part will contain the "laws relative to members of the medical profession in particular, viz. their qualifications, rights, privileges, duties, and liabilities, whether as physicians, surgeons, apothecaries, &c." Now as this fourth part will be included in the second volume, which will be published separately, it appears to us that he intends and expects that lawyers will be the chief purchasers of the first volume, (now under review,) and medical practitioners of the second. We would advise Mr. Chitty, therefore, to vary the amount of the editions according to this calculation. Mr. Chitty informs us that, in the *fifth* part of the work, that is, in the second volume, will be introduced the subject of *medical evidence*, and here he lets fall a sentiment which we have always advocated, namely, that the best way to study the law of *medical evidence*, is to study our own profession thoroughly. He does not agree with that amiable but enthusiastic writer, the late Dr. J. Gordon Smith, that "medical evidence" should be made a kind of distinct study, and that the individuals who are to give testimony in a court of justice, should agree previously, as to the tendency of their answers. "Lastly is given a view of *medical evidence*, and this rather to *limit* any direct professional education as regards the *manner* of giving evidence, and to suggest the higher importance of each individual, stating his *own genuine testimony*, rather than studying to agree with other practitioners."

Our author dwells with great reason and force of argument, on the necessity of a general knowledge of medical jurisprudence on the behalf of members of the Legislature, who are often called upon to enact laws relating to public health, nuisances, quarantine, &c. &c. He aptly asks, would members of the Legislature make the crime of procuring abortion depend on the period of quickening, if they knew any thing of the laws of fœtal life? Or would they be so ready to put quarantine in force, if they were better acquainted with the laws of epidemic diseases? The late choleraphobia, and the impotent attempts of legislators to arrest the current of epidemic constitutions of the atmosphere afford biting examples of the truth of Mr. Chitty's remarks.

That all persons acting in a judicial capacity, should have such a general knowledge of medicine, as to enable them to comprehend the import of the chief technical terms employed by medical men, is strongly insisted on by our author. He illustrates this in the following manner.

"Suppose a medical witness should swear that a blow or wound had been given on the Clavicle (vulgarly called the Collar-bone,) or the Scapula (vulgo Shoulder-blade), or in any other technically described part of the body, would it not be desirable that the Judge should instantly, without stopping to inquire, not only know the name of the part affected, but also be able to judge for himself what would be the probable consequences of the injury; for otherwise his mind would be occupied in seeking information merely on the import of terms, or in explanation of probable results, when he should be proceeding with more important considerations; and few minds are so powerful as to be able simultaneously efficiently to consider two different subjects."

Mr. Chitty quotes an observation from Professor Amos, which we hope will not be lost on judges and counsellors. That gentleman remarks that "even some judges as well as counsel are not unfrequently *very shallow men in science*, and therefore too apt to attack medical witnesses for using technical terms unknown to them—a just reflection, at least, upon counsel, that it is to be hoped will, ere long, be shewn to be no longer well-founded." This state of things, however, we can hardly expect, in our days. It cannot be expected that lawyers who have so much to study on their own account, should be very conversant with medical technicalities, and therefore it is the duty of the professional witness to avoid such terms as much as possible. We have lately had a lamentable instance of want of tact, and even of common sense, exhibited in the committee on medical education in the House of Commons. A physician, of no small calibre, and who entertains no mean opinion of himself, on being asked if the limits between physic and surgery were so well defined as to require a distinctly different education for each kind of practitioner, replied, in about one hour's dissertation on the anatomy of the eye—on the nature of iritis—on the modus operandi of mercury, and other matters which were about as relevant to the question put to him, as a treatise on electricity is to the manufacture of soap. During this exhibition the whole audience were laughing or grieving at the folly of the exhibitor, who construed the universal action of the risible muscles into rapturous plaudits for the immense knowledge which he was pouring forth to enlighten the world!! A more sickening specimen of anile imbecility was never seen upon earth; and it will form a blot on the records of the important investigation alluded to, which the FELLOWS of the College may very fairly throw in the teeth of the Licentiates, as a precious specimen of their order. It is impossible, indeed, to frequent our courts of justice, without daily witnessing the most lamentable examples of want of tact, and common judgment on the part of medical witnesses. This is remarked by the bar, the members of which speak in derision of the testimony of professional men. These hints will not, we hope, be lost on our brethren.

In respect to barristers, a general idea of medical matters is essential to their conducting many important causes at the Bar.

"With respect to *Barristers*, or at least all who practise at the Criminal Bar, or hold a brief in a life policy cause, or in an action against a surgeon for alleged misconduct, it is as much their professional duty as their interest to obtain a complete and perfect knowledge of every branch of these subjects so intimately interwoven with each other. How disastrous to his credit, and how painful to his feelings, would it be to hear it asserted, after the execution of a prisoner, that the conviction was attributable to his Counsel not having put, or having injudiciously put, a particular medical question, or generally proper questions connected with the subject; and yet such omission or blunder might have been probably attributable to mere ignorance of subjects which were not directly connected with the more ordinary and daily object of his *legal* study."*

---

"* In Mary Wright's case, convicted at the Spring Norwich Assizes, A. D. 1833, of murdering her husband, the counsel, Mr. J. Sidney Taylor, so ably

In advocating the propriety—as well as the possibility of a lawyer becoming generally acquainted with the principal points of medicine, and that without neglect of their own proper studies, Mr. Chitty quotes some observations from Dr. Adam Smith, respecting apprenticeships, which we hope will, ere long, be acted on by our legislature, as far as the medical profession is concerned. "Dr. A. Smith observes, there is no necessity for an apprenticeship of *seven years*, exclusively devoted to watch-making, or any other department; nor is there any occasion for the waste of *seven* years—and still more of *ten* years, upon the sole study of the dead languages. Much of the time ought to be occupied in the examination of other branches of science and literature, and which would infinitely more interest and improve the minds of youth, and enlarge their intellectual powers and attainments." These truths would be more useful than acceptable at Oxford, where so many of the best years of life are occupied with classical lore—or rather with acquiring the meaning of words, instead of cultivating those arts and sciences which prove useful through life. Cambridge, where mathematics are more favoured, does not fall so much under the scope of this censure as the Sister University. The following perhaps is modestly expressed.

"With respect to *Medical Practitioners*, although I cannot anticipate that my observations upon *Medical* subjects will be regarded as of any authority, yet as respects *Students* in Medicine or Surgery, I venture to hope that the analytical summary and condensed view of most of the present doctrines may be found useful; at all events, as regards the former, it is of the utmost importance that they should be fully informed of *the laws* respecting all injuries by violence, poison, or otherwise, to the person, and many other branches of the law, and which have been greatly, and in many respects entirely altered since the publication of the previous treatises upon Medical Jurisprudence. It is therefore hoped that the work may be found useful to *them*, as affording a view of the *law*, to which their attention and testimony is to give due effect; and the pages respecting the peculiar *laws* affecting the *Medical Profession* and its branches, may be found important even to their pecuniary interests and welfare."

If this "analytical summary and condensed view" of the present doctrines have been drawn up by a lawyer, it is one of the cleverest compendiums we have ever seen. It is worth five dozen of the compendiums of Thomas, Reece, Graham, and other compilers of popular systems of medicine. We may here, also, take occasion to correct an error which has been promulgated by some of our contemporaries, namely, that this first part is only a fifth part of the whole. The other four parts are to be comprised in a volume equal in size to the present part, and thus the whole expense will be two guineas instead of five. We conclude, then, with a confident expression that this is the best compendium of medicine that has ever been drawn up for the benefit of non-medical readers.

---

drew the attention of the learned Judge to the absurdity of executing a woman with child, though *not quick*, that he contributed towards saving her life; and after a jury of matrons had, upon her plea of *pregnancy* in delay of execution, *erroneously* found her not to be quick with child, she was delivered of a mature child at a time which clearly denoted that she must have been quick at the time of the trial, and the woman was finally transported instead of executed."

---

### The Otic Ganglion.

In the Number of this Journal for April, we presented to our readers a short account of the difference of opinion existing amongst the anatomists of Germany, as to there being such a ganglion as that described by Arnold, under the name of the otic or auricular ganglion; and we concluded by observing that, in this country, Mr. Mayo had recently acknowledged its existence. We were not then aware that, in doing so, we scarcely acted impartially, in omitting all mention of the share which Mr. Thurnam (at that time a pupil at the Westminster Hospital, and secretary to the Westminster Medical Society) had in its recognition. We are, therefore, inclined to lay before our readers a little sketch of the history of this ganglion.

The ganglion oticum, seu auriculare, called also ganglion Arnoldi by Professor Tiedemann, in honor of its discoverer, Dr. Frederick Arnold, of Heidelberg University, was described by that anatomist, in his work on "the Cephalic Portion of the Vegetative Nervous System in Man," which was published at Heidelberg and Leipsic in 1831. A translation from this work, of his description of its anatomical and physiological relations, by Mr. Tybe, of Devizes, will be found in the Edinburgh Medical and Surgical Journal for July, 1833. In the following August, Professor Mayo, in a paper entitled "Physiological Observations," published in the London Medical Gazette (vide No. 45), stated that himself and Mr. Partridge had dissected for it in two subjects, and had been unable to discover it. He, therefore, concluded, that Arnold was mistaken as to the existence of any such ganglion, and stated his belief, that the nerves which he describes as peculiar to it, really belong to the third division of the fifth pair itself. Matters stood thus, when, as we find from the Medical Gazette for Dec. 21st, 1833, Mr. Thurnam commenced his dissections, and, having made out its existence in three instances, he announced this, as well as his mode of dissection, in the above Journal.

A month after this, Mr. Mayo, in the same Journal,* acknowledged its existence, and the correctness of Mr. Thurnam's method of dissection; and also announced the existence of filaments, not described by Arnold, which establish a connexion with the chorda tympani.

* January 18th, 1834.

We have seen Mr. Thurnam's preparations of the ganglion, and have compared them with Arnold's figure, with which they appear accurately to correspond, except in the shape of the ganglion, which is not of so regularly ovoid a form as he has represented it, but more semilunar, as stated by Mr. Mayo.

The interesting nature of the anatomical relations of this ganglion to the organ of hearing, indicating a corresponding physiological relation, and such an one as the ophthalmic ganglion bears to the eye, as Arnold has so beautifully pointed out, induces us to consider it as an important discovery in the anatomy of the nervous system of "organic life."

---

AN ESSAY ON THE RELATION OF THE THEORY OF MORALS TO INSANITY. By T. MAYO, M.D. Octavo, pp. 49. 1834.

This subject borders a good deal on the metaphysical, and we apprehend that it will not excite much attention among the author's professional brethren.

Dr. Mayo appears to think that, speaking generally, there is placed within the mind of man a monitor, called CONSCIENCE—a sense of moral feeling —which tells him when he does wrong —but that there are some exceptions, where this same conscience either does not exist at all, or is overpowered by their vicious propensities, and kept in abeyance. But these rare exceptions he considers as only proving the general rule, and he triumphantly asks Dr. A. Smith *(without fear of an answer)*: "What is *that* in which *all* men sympathise, as obligatory on their moral conduct?" As Dr. A. Smith is not likely to hear the question, we will endeavour to answer for him. Our reply is—"NOTHING." There is *nothing* in which *all* men sympathize as obligatory on their moral conduct. If there was, the Chinese would not fling their offspring into the river without any qualm of conscience, while the Englishman acknowledges it a crime against God and man, and would swing for it at Newgate. If there was an innate, congenital, and universal monitor, or conscience, independent of education, the Hindoo would not hold it a crime to slaughter a cow on the banks of the Ganges, while the cockney looked upon it as no sin at all in Smithfield. Where is this innate monitor, when the Otaheitan slaughters and eats his enemy, at which the conscience of the Christian revolts? But why need we multiply examples. It is as clear as the sun at noon-day, that to the immortal soul is given an understanding, by which it learns good and evil according as it is taught. It has no innate power or conscience, by which it can discriminate good from evil, independent of education. If there was such a power, what need had we of revealed religion, to direct us to the one, and to guard us against the other? If there was an innate power or conscience, there could be no exceptions. Our author, however, thinks that there are such exceptions, and that the individuals thus excepted from the influence of conscience should be treated as insane. The following passage will illustrate Dr. Mayo's notions on this point.

"Mr. A. was born in a respectable station, and is in possession of a good fortune, of as much, at least, as he has allowed to remain out of a good fortune. He has a wife and children, and as many friends, or rather associates, as his convivial qualities retain for him, in spite of the hardness of his character. He has always been profusely extravagant; for his passions and appetites have compelled him to squander money, which he would probably have hoarded, if his selfishness had taken that turn. His temper is at once stern and violent; and all who know him expect that the dispositions of his will must prove him, to the last moment of his life, utterly unjust. If he had sufficient courage he would rob or murder; for his cupidity is under no moral check; but he is naturally very timid, and owes to this circumstance his freedom from such acts as the law construes into crimes. Such is Mr. A., and such also, or as nearly such, as the distinctive points which separate all individuals will allow, is Mr. G. his neighbour. But there happens to exist a peculiarity in the latter, which materially alters the course of his life, and its results upon others. Mr. G. was observed to talk very much to himself. This excited attention; and, on further inquiry, it was discovered that he was habitually under the influence of false perceptions, and that he considered himself solicited by certain voices, audible only to himself, to perform those actions which indeed flowed naturally enough from his own evil dispositions. Mr. G. was accordingly recognized as a lunatic, and placed under restraint. Thus the family of Mr. G. is secured against the results of his moral character; and his fortune will descend according to the principles of law; thus dealing out a justice to others, and a protection to *him*, which would have been refused, but for this hallucination."

We certainly think it would be very desirable, if every man who is in the habit of injuring his family, or even himself, by his vices, his follies, or his extravagances could be checked; but then, to decide such knotty points of moral conduct, and to measure with exactness the length and breadth of a man's *conscience*, we must have a jury of moral philosophers and metaphysicians, with Dr. Mayo as the foreman—for we are quite sure that no common or special jury would be able to decide such questions.

Dr. M. proceeds to inquire into the analogy which this "moral insanity" bears to the commonly recognized, or "intellectual insanity." The following passage embodies this analogy, and also gives us a definition of insanity—a thing very much needed in the present state of our knowledge.

"Both perception and reflection in the insane intellect are governed by laws absolutely different from those which are recognized by the common perceptions of mankind. This, however, happens also to persons whom it would be absurd to call insane, or even eccentric. Thus a person under an excited state of mind, or in ill health, sees visions and hears voices equally foreign to the common perceptions of mankind with those of the lunatic. The difference between the insane and all others who have similarly erroneous perceptions, is, that the latter are possessed of a power by which they can question the justness of ther own perceptions, the insane have no such power; for when they begin to make use of such a power, they are so far convalescent. But as the erroneous notions which the insane intellect forms respecting the objects of sight cannot be referred by it by any test of their soundness, as possessed by it in common with the rest of mankind; so the conduct suggested by the inclinations and tendencies of the *vicious* in the sense in which we are now estimating vice, cannot receive any modification from a reference to a moral sense or common feeling of approbation. The analogy may be pursued further: for persons thus destitute of the moral sense may, from the very absence of a conflicting principle such as it would supply, move much more smoothly through their course of wickedness, than criminals of an opposite kind, namely, those who do wrong, 'having a law in themselves, their consciences bearing witness, and their thoughts accusing them;' and so also the progress through life of persons intellectually insane, is often very quiet and regular, far more so, at least, than that of many persons justly considered sane, but exposed, from the very struggles of their reason with antagonist principles of the intellect, to constant uncertainties and doubts, which the insane are exempt from. Many a person, indeed,

'Cui sic absumpta voluptas
Et demtus per vim mentis gratissimus error,'

may envy the contented and self-satisfied lunatic.

Without supposing myself to have exhausted the points of analogy between these states of moral and intellectual deficiency, enough has, I think, been adduced to warrant our assuming, that the absence of the moral sense constitutes a form of unsoundness, analogous to that intellectual unsoundness, which is commonly understood when the term insanity is used; and, accordingly, that we may talk of a moral and of an intellectual insanity as contradistinguished species."

Dr. Mayo asks, is it not probable that the same treatment may be applicable to the moral depravity as to the mental derangement? We think that, as far as *prevention* goes (which is said to be better than cure), a cell in a madhouse would very effectually prevent the commission of crimes—and especially the extravagance of spendthrifts; but if this kind of moral insanity were to come under the law, where, in God's name, would we find prisons? Well may Dr. Mayo say, "that the difficulty of finding that point, beyond which *vice* may be considered as the subject of coercion, on the ground of unsoundness, will necessarily be great." A little farther on our author remarks—"the above considerations may appear to many persons absolutely erroneous: others may think them theoretically correct, but impracticable." We acknowledge that we are among the latter. For, even if the suggestion of Dr. Mayo, that common juries should be superseded by scientific, were put in practice, we are quite confident that twelve doctors would not be brought to agree upon any one case of moral delinquency, unless there were symptoms of common or intellectual insanity present. Dr. Mayo is an excellent physician, and, we have no doubt, a very clever and subtle metaphysician; but we suspect that, like an illustrious statesman and philosopher of this country, he is—

"Too fond of the right to pursue the expedient,"

and perhaps addicted, like the same personage, to—"cut blocks with a razor."

---

### Climate of the Lands End.

From a very able and elaborate paper on the medical topography of the hundred of Penwith, district of the Lands-end, by Dr. Forbes, of Chichester, and published in the transactions of the Provincial Medical Association, we extract a passage or two respecting the climate of Cornwall.

The whole district, in question, possesses, as far as regards climate, all the advantages and disadvantages of a small island, moderately elevated above the level of the sea, and placed at a distance of forty or fifty miles to the westward of the most southerly point of the mainland. In stating the various meteorological results, Dr. Forbes places, in most instances, the climate of London in opposition, that locality being best known, and affording a fair specimen of the mean climate of the southern part of England.

" 1.—*Annual Mean Temperature.*

| | |
|---|---|
| Landsend (Penzance)................ | 51.8° |
| London............................ | 50.3° |

2.—*Mean Temperature of the Seasons.**

| | *Winter.* | *Spring.* | *Summer.* | *Autumn.* |
|---|---|---|---|---|
| Landsend.... | 44° | 49.6° | 60.2° | 53.3° |
| London ..... | 39.1° | 48.7° | 62.3° | 51.3° |

3.—*Mean temperature of the Months.*

| | *Jan.* | *Feb.* | *Mar.* | *Apr.* | *May* | *June.* | *July.* | *Aug.* | *Sep.* | *Oct.* | *Nov.* | *Dec.* |
|---|---|---|---|---|---|---|---|---|---|---|---|---|
| Landsend, | 42.5 | 43.5 | 46.4 | 48.5 | 54.0 | 58.5 | 61.2 | 60.9 | 57.6 | 53.7 | 48.8 | 46.1 |
| London.. | 37.3 | 40.4 | 42.6 | 48.0 | 55.6 | 60.0 | 63.4 | 63.5 | 58.8 | 51.7 | 43.4 | 39.5 |

4.—*Difference of the Mean Temperature of Winter and Summer.*

| | |
|---|---|
| Landsend.......................... | 16.2° |
| London ........................... | 23.2 |

5.—*Difference of the Mean Temperature of the warmest and coldest months.*

| | |
|---|---|
| Landsend .......................... | 18.7 |
| London ............................ | 26.2 |

6.—*Difference of the Mean Temperature of successive Seasons.*

| | *Of Winter & Spring.* | *Of Spr. & Sum.* | *Of Sum. & Aut.* | *Of Aut. & Winter.* |
|---|---|---|---|---|
| Landsend...... | 5.6° | 10.6° | 6.9° | 9.3° |
| London ....... | 9.6° | 13.5° | 11.0° | 12.2° |

7.—*Difference of the Mean Temperature of successive Months.*

| | *Jan. & Feb.* | *Feb. & Mar.* | *Mar. & Apr.* | *Apr. & May.* | *May & June.* | *June & July.* | *July & Aug.* | *Aug. & Sep.* | *Sep. & Oct.* | *Oct. & Nov.* | *Nov. & Dec.* | *Dec. & Jan.* | *Mean* |
|---|---|---|---|---|---|---|---|---|---|---|---|---|---|
| Landsend, | 1.0 | 2.9 | 2.1 | 5.5 | 4.5 | 2.7 | 0.3 | 3.3 | 3.9 | 4.9 | 2.7 | 3.6 | 3.1 |
| London.. | 3.3 | 2.2 | 5.3 | 7.6 | 4.3 | 3.4 | 0.1 | 4.7 | 7.0 | 8.3 | 3.8 | 2.2 | 4.3 |

8.—*Absolute Range of the Thermometer.*

| | *Highest Extreme.* | *Lowest Extreme.* | *Greatest Range.* |
|---|---|---|---|
| Landsend, (12 years,).... | 84° | 19° | 65° |
| London, (30 years,)...... | 96° | 5° | 91° |

9.—*Mean Range of the Thermometer.*

| | *Annual Range.* | *Monthly Range* | *Daily Range.* |
|---|---|---|---|
| Landsend ............... | 49° | 24° | 6.7° |
| London................. | 64° | 34° | 11.8° |

* The more common divisions of the seasons is here adopted, *winter* comprehending Dec. Jan. Feb. and so on.

10.—*Variation of Temperature of successive Days for the whole Year.*

| | *Mean Variation.* | *Extreme Variation.* |
|---|---|---|
| Landsend ................ | 2.6° | 10° |
| London.................. | 4° | 18° |

The preceding results place, in a striking point of view, the great peculiarity of the climate of this district, as to temperature: it possesses, as has been already remarked, all the habitudes of a small island remote from any large continent: it exhibits, in a great measure, the qualities of what may be called the *ocean climate.* This is characterised essentially by a remarkable degree of *equability* of temperature or small extent of range above or below the mean. In this respect the district of the Landsend is superior to any other part of England, and, indeed, to any place in Europe of which we possess meteorological accounts. Madeira is the only climate which Dr. Clark considers as superior in regard to equability of temperature.*

As necessary consequences of the greater equability, it will be seen by the preceding statements that, although there is a difference of only 1½° between the mean annual temperature of this district and that of London, there is a remarkable discrepancy between the different seasons at the two places. Thus it is seen that while the summer temperature of the district is rather more than 2° *lower* than that of London, the winter temperature is very nearly 5° *higher.* The spring temperature of both places differs less than one degree, and that of autumn is 2° in favour of the Landsend. It thus appears that while the winter is much *warmer* than that of London, the summer is considerably *cooler;* the autumn is as much warmer as the summer is cooler, while the spring is nearly equal at both places. The temperature of the whole spring at Penzance, as taken by the thermometer, is, indeed, rather higher than that of London; still it will be observed, on referring to the tables, that this superiority is derived almost entirely from the month of March, the temperature of April being very nearly the same in both places, while that of May is more than a degree and a half *lower* at the Landsend.

The singularly small range of the thermometer in this district, gives rise to a remarkable difference in the relation of the temperature of the day and night, here and in some inland places; in summer the nights being as much warmer as the days are cooler, and in the winter both the nights and days being warmer. This will appear in a striking manner by comparing together the mean of the extremes, i. e. of the highest and lowest degrees of temperature, as shown by the register thermometer during the two seasons, at London and Penzance.

| | *Summer.* | | *Winter.* | |
|---|---|---|---|---|
| | Day. | Night. | Day. | Night. |
| London............ | 81° | 42° | 52° | 23° |
| Penzance .......... | 72° | 49° | 55° | 31° |
| London higher...... | 9° | — | — | — |
| Penzance higher .... | — | 7° | 3° | 7°." |

It appears that, for equability of temperature, there is no place in England equal to the Landsend, excepting, perhaps, the neighbouring isles of Scilly. But it must be remembered that temperature alone is not the grand point of medical topography. The moisture of the climate must be taken into consideration—and even the *gloominess,* as acting on the spirits and health. We apprehend that Penzance, so much lauded a few years ago, will not continue a favourite residence for pulmonary invalids, on account of the fogs and rains, the dreary shores, and the treeless hills of Cornwall. The cheerfulness of Hastings or the Isle of Wight, is no trifling advantage, where the mind is borne down by the infirmities of the body.

* Influence of Climate, p. 63.

### Mr. Sandwith on the Nature and Treatment of Whooping-Cough.

In a late Number of Dr. Hunter Lane's Monthly Archives, there is a paper of much research and practical discrimination on the above subject, from the pen of Mr. Sandwith, of Bridlington, who witnessed an epidemic, in the year 1830, of hooping-cough. In the majority of cases that presented themselves to Mr. S. there were evidences of acute or subacute inflammation. In the latter cases, the disease was greatly moderated by leeches to the region of thyroid cartilage, as recommended by Dr. Dawson. When the head was affected, the leeches were best applied between the shoulder-blades, with calomel, pulv. Jacobi, and the usual adjuvants. " Several examples of the disease, however, in its more exquisite forms, demanded more energetic means to subdue the inflammation. In some of these cases, the child lay constantly struggling under the aggravated dyspnœa of acute bronchitis, that " permanent difficulty of breathing, which continues between the coughing fits," as described by Darwin. As might be expected, there was considerable mortality where professional aid had not been sought.

In these cases, it was necessary to combine local and general bleeding with emetics, the warm bath, calomel purgatives, and blisters. The strong tendency to cerebral congestion deterred from the use of opium, in the form of Dover's powder, to any extent. The following case affords a striking example of metastasis from the mucous membranes of the brain.

" Master S. aged 4, became affected by hooping-cough in May. The attack was severe, but without producing acute symptoms of bronchitis ; emetics, purgatives, and the prussic acid made no decided impression on the complaint. After exposure to cold, in the latter end of June, his cough suddenly left him ; and general lassitude, loss of appetite, and increased feverishness supervened. The fever, with symptoms of cerebral irritation, increased from the 24th to the 30th, when actual phrenitis demanded more active treatment than the less violent anti-inflammatory remedies hitherto used. About a teacupful of blood was taken from the arm on that day, and seconded by leeches and free purgation. The general bleeding was repeated on the first of July, and leeches to the temples and the sides of the neck were afterwards several times freely employed. Sleeplessness, and that delirious anguish which mark cerebral inflammation in children, were prominent and distressing features during the severity of the attack; there was also, for a day or two, rigidity of the spinal muscles, and a peculiar drooping of the head to the left side, with a semi-rotatory motion. The symptoms of cerebral irritation, though moderated by active treatment, did not wholly decline before a fortnight had elapsed from the time of the first general bleeding. The emaciation was equal to what we observe in the most protracted cases of fever. The first clear proof of the entire removal of cerebral irritation was a return of the cough, with its distinctive hoop,* which continued in a mild form during the whole period of convalescence. Natural and refreshing sleep, at first excessively profound, succeeded to the long-continued scenes of excitement. A change of air finally removed the cough, and asses' milk repaired the wastes of the system. The case just described illustrates the truth of Dr. Palmer's remark relative to the complications of hooping-cough, that they ' will demand, with great vigilance and energy, a variation of practice precisely corresponding with such change."

---

### The Morbus Oryzeus.

Our readers are aware with what un-

---

* " An interesting case of hooping-cough alternating with measles, and re-appearing on the decline of the latter, is related by Dr. Ferriar.—*Vide Medical Histories and Reflections,* vol. iii. p. 217."

shaken constancy Dr. Tytler has cleaved to the doctrine, of the epidemic cholera arising from the use of damaged rice. Through good and through bad report, in spite of argument, and in defiance of fact, the Doctor has hugged his bags of rice; and, holding them up to the attention of the world and of the Medical Society, he has endeavoured to seal, with philanthropic fury, the lid of this new Pandora's box. These degenerate times can scarcely afford one other specimen of such single-minded, and, indeed, single-handed, advocacy of a cause, which is derided by the ignorant million.

Towards the latter end of this month of July, 1834, the profession became aware, and the newspapers informed the public, that some fatal cases of cholera had occurred, while others of extreme severity were daily occurring. Then was the good Doctor up and stirring, and, like that honest animal whom the Lake Poet has described as sagaciously "turning his left ear round on the pivot of his skull," our watchful hero listened for the approach of the dreaded rice. That he heard it coming we will not say—but that he saw it will speedily be shewn.

Great minds are always in advance of their century. They pursue untrodden tracks, and detect the most important and extraordinary truths amidst rubbish neglected by vulgar observation. It is a trite remark, that while Sir Isaac Newton discovered the laws of gravitation, from watching the fall of an apple on the ground, the majority of men would have picked it up and eat it. Dr. Tytler is one of those commanding intellects that disdain the processes of ordinary reason. When he found that the cholera was coming, or come, he did not pursue its cause in the air, or in the water—in the heat or in the cold—in the diet or in the clothing—in contagion, or in epidemic influence—he found it in the *London Price Current of June*. His well-trained nose scented out the commercial iniquity which had sacrificed, and, but for Dr. Tytler, would continue to sacrifice its victims. Full of this great discovery, he immediately communicated it to the Lancet, in which it was greeted with a cordial grasp. That Journal observed, on the interesting occasion, that "the question is one of prodigious importance to the community, and that Dr. Tytler is indebted to its gratitude for the zeal and ability he has displayed in introducing the subject to general notice."

We must now let Dr. Tytler speak for himself, and lay before our readers the *London Price Current*, in its native and beastly wickedness.

"Should it unhappily prove eventually," says Dr. Tytler, "to be the fact that *Asiatic Cholera* or *Morbus Oryzeus*, has re-appeared *in this City*, or in any other part of Great Britain and Ireland, the following extract from the *London Price Current* of June 3d, 1834, will satisfactorily explain the cause of this unfortunate circumstance, and abundantly prove that until vigorous measures be adopted to expel *poisonous rice* from the markets of England, the health of the community cannot be considered as secure from this terrible scourge and destroyer of the human race.

'RICE.—Large parcels of rice continue to be offered at public sale, which enables the buyers to purchase BY PRIVATE CONTRACT AT VERY LOW PRICES; Patna kind has been sold 12*s*. 6*d*. to 13*s*.; ordinary white Bengal at 10*s*. 6*d*. By public sale this day, 2970 Bags Rice; a part sold rather higher; the usual buyers are now attracted to Rice, *on account of the reported short Corn Crops*.'—From the *London New Price Current* of Tuesday, June 3d, 1834."

We cannot contemplate this cold-blooded traffic in pestilence, without a shudder. What punishment can be too great for the miscreants who can deliberately barter in "Patna kind," and can coolly sell "ordinary white Bengal at 10*s*. 6*d*." We appeal to the Legislature, to deal with this new and monstrous guilt as it deserves; and should the people's representatives be callous to our prayer, we adjure the people to take vengeance on the criminals, and hurl the rice-contractors and their rice-bags into the Atlantic. For Dr. Tytler, money were an inadequate reward. He will live in the gratitude of his Country, and the Poet Laureate may be invited to immortalize him in a mighty Oryzean Hymn, commencing, perhaps, with—

*Risum* teneatis amici.

---

### Perforation of Strictures.

Mr. Stafford has offered some desultory observations and published twenty-one circumstantial cases, to prove the necessity, the propriety, and the success of perforating strictures of the urethra.

When we take into account the numerous circumstances which impair the judgment, even when they do not affect the honour of those whose practice is novel or peculiar, we must cease to entertain surprise at the scepticism extended to the facts which such reasoners adduce. The amount of that scepticism may possibly be unjust, yet they who are most familiar with the history, and indeed with the practice of medicine, are usually found to entertain it most freely. The rise and fall of doctrines and of methods are too rapidly displayed to allow it to subside.

The preceding observations are, perhaps, inapplicable to the instance of Mr. Stafford. His honour cannot be questioned, his judgment may not be impugned. Yet some objections to his cases and his practice present themselves, and his obvious inclination to pursue and to elicit truth, will probably induce him to regard them with attention, if not with favour. Mr. Stafford's activity and zeal have been proved by his several contributions to literature and science. He has printed and published a copious pamphlet, on the application of wax to ulcers; and although the former has been little read, and the latter has scarcely been found to succeed, the labour of the composition and the ingenuity of the proposal continue undiminished. He has also published a lengthened work on Diseases of the Spine, the merit of which has been thought by some to be overbalanced by its bulk. But Mr. Stafford's perforation of strictures has attracted and received the attention of the public, to an extent much greater than his other attempts to improve the science and details of surgery.

We have stated that Mr. Stafford has published the particulars of twenty-one cases in which he divided urethral strictures by means of the lancetted stilette. The experienced surgeon involuntarily starts, and inquires how Mr. Stafford could have seen so many instances, in which it was impossible to introduce a bougie or catheter, and to carry it through the strictured part. The cases, in public and in private practice, in which this impossibility is experienced by those possessed of moderate dexterity, and acquainted with the best modes of treating stricture, are so few, so very few, that surprise at the good or evil fortune of Mr. Stafford is necessarily great. We lately heard Mr. Brodie declare, that for many years he has been foiled but once in getting an instrument of some description through the stricture. Perhaps other surgeons have not been so fortunate, yet we feel convinced that all will be astonished at the numerous array of impassable strictures displayed by Mr. Stafford. In our own experience, we have generally found that the number of unsuccessful cases afforded a tolerably just criterion of what was *not* the adroitness of the surgeon. On many occasions, patients present themselves to the house-surgeon or the surgeon of a hospital, declaring that they labour under strictures, for which no instrument could be passed by the various medical men who have attended them. Yet in the vast majority of such cases, instruments are introduced at the institutions to which the individuals apply. Did the record of these cases attest or confirm the superior efficacy of a particular method, it would probably be easy to adduce as long and as imposing a list on the side of dilatation as Mr. Stafford has advanced in favour of division.

It may be stated as a general maxim in surgery, that of two kinds of practice equally efficient, the mildest, that is, the least painful and least dangerous, should always be preferred. Mr. Stafford asserts that he has never known mischief arise from the operation of division, and that "the pain has been but trifling, not more, nor even in some cases so much as the puncture made in bleeding." This is an encouraging picture it is true, but the surgeon who has witnessed the pain occasionally caused by the gentlest use of the bougie, must hesitate to place implicit faith in the declaration, that the employment of a cutting instrument is attended with so small an amount of suffering.* Some allowance must, perhaps, be made for the natural fondness for our offspring, exhibited in the scientific world, as well as in the animal, a fondness which might have influenced, and may reasonably excuse the apparent partialities of Mr. Stafford. Those partialities are great and generous. He affirms, for example, that "no false passages have been made" by division. It might be difficult, on physical principles, to demonstrate, that an instrument like a lancetted stilette, which cannot from its constitution be guided with even so much precision as a bougie, and which must necessarily penetrate through all opposed to it; it might be difficult, we say, to demonstrate that such an instrument would not be likely to occasion a false passage. Yet this instance exhibits the superiority of experience to the efforts of reasoning. Whatever might have been anticipated, Mr. Stafford assures us that no false passage has been made; and that declaration must quiet the captious or presumptuous disputant. The happy picture displays no contrast of light and shade—it is sunny and clear as an Italian sky. Mr. Stafford thus closes his paper in our contemporary; the passage being perhaps more decisive of his good fortune, than characteristic of his attention to grammatical construction.

"Nor indeed has there *been* in any instance a single unfavourable symptom *occur*.† The treatment has been always attended with success, and the patients, from having been in the most deplorable and painful state of disease, being emaciated and worn out by their long and continued sufferings, have in a short period been so relieved that they have gained flesh, and have become healthy and strong."

This flattering picture might almost appear to silence objection, yet we candidly confess that it would not induce us to resort to division, until we had practically satisfied *ourselves* of the impossibility of passing a catgut or a wax bougie, or catheter. We cannot avoid entertaining the suspicion that in some of the cases detailed by Mr. Stafford, such a proceeding, if conducted with caution and adroitness, would, perhaps, have been attended with success. The unnecessary employment of violence or of the knife is always to be deprecated, because it creates, or, at all events, increases the natural prejudice against surgical operations, and may tend to prevent some persons from submitting to them, whose cases require and whose lives might be preserved by them. We are disposed to believe that the division of strictures is too much praised and too much practised by Mr. Stafford.

Yet that gentleman, not content with his triumph over the urethra, has carried his views of conquest to the rectum. In the number of the Medical Gazette for July 26, of the present year, Mr. Stafford inserts a description and a drawing of some instruments invented or employed for the purpose of dividing what he terms an *elastic stricture*. This is a stricture of the permanent kind, which will admit through it a bougie, but which, immediately on its withdrawal, will retract to its former calibre.

"These instruments resemble in their structure the lancetted stilettes, excepting that, instead of a lancet being thrust out at the point, a semilunar blade comes out at the extremity on the side, above, below, or laterally, as may be

---

* We lately witnessed an attempt to divide a stricture with a lancetted stilette. The patient writhed and shouted with the pain.

† It would not be easy to decide in what classic work or in what society this species of construction may be met with.—*Rev.*

required, and as represented in the engraving. The mode of using them is to pass them through the stricture, and then throw out the blade, and draw the instrument back again through the contracted part until it is divided, when the blade is allowed to retire into its sheath."

An instrument of this description is applicable, according to the account of Mr. Stafford, to division or slitting of an enlarged middle lobe of the prostate gland—to the division of stricture of the œsophagus—and to that of stricture of the rectum.

Mr. Stafford relates at some length, a case of stricture of the rectum in which he performed division of the stricture. The stricture is said to have been situated at the distance of two inches and a half from the anus; it appeared to be indurated, and would allow a No. 12 urethra bougie to pass through it. On the 25th of February Mr. Stafford divided the stricture opposite the sacrum with an instrument such as has been mentioned. Tenderness of the abdomen appeared next day, and continued at intervals till the 8th of March. From that time till the 20th she did pretty well, but on the latter day she was attacked with erysipelas of the head and face, of which she died upon the 25th. We should state that, previous to her death, "a large bougie" could be passed with ease and with little pain.

Mr. Stafford's account of the dissection is imperfect. He describes the condition of the brain and of its membranes—a tuberculated liver—traces of peritonitis—cicatrized ulcers of the mucous membrane of the small and of the large intestines—ulcerations, not cicatrized, in the sigmoid flexure of the latter. But where some prolixity might be excused—in the details of the nature of the stricture—curiosity is baffled by a meagre memorandum. "The gut at the strictured part," says Mr. Stafford, "would admit through it a bougie as large as the thumb. The cicatrix of the wound was very perceptible, and it was covered by a membrane analogous to that lining the intestine."

We may be wrong, and Mr. Stafford may be right, but we do conceive that cases so related are of little essential benefit to science, whatever they may be calculated to confer on the narrator. Mr. Stafford may suppose that had the patient not died accidentally of erysipelas, she would probably have recovered, but we do not perceive as he appears to do, that the dissection absolutely proves this probability. To enable the reader to form an opinion, he should be made acquainted with the nature of the stricture. The pathologist perhaps will be tempted to smile at the sanguine therapeutics of our author. He (the author) attributes the cicatrization of the ulcers of the mucous membrane of the colon to his operation on the rectum, and concludes that all would have healed from the same influence. The reader of the case will perceive that symptoms of abdominal inflammation immediately followed the division of the stricture, and continued for ten or twelve days after its performance. Their cessation is converted, by the sanguine pathology of Mr. Stafford, into a proof that the ulceration of the bowels would have healed. "That such an inference (the ultimate healing of all the ulcerations) may be drawn is further proved by the pain and tenderness in the abdomen having ceased; shewing that the inflammation had diminished, and all the other symptoms abated." Most candid reasoners would probably attribute those inflammatory symptoms to the performance of the operation.

We would not be deemed hypercritical, nor would we be willingly accused, with justice, of endeavouring to pull down the merit of any man. But we really think that Mr. Stafford is inclined to carry his love and his advocacy of division of strictures too far, and admitting, as we do, the occasional utility and even the necessity of such an operation, we feel disposed to warn the inexperienced part of the profession against its too general and indiscriminate employment.

### Dr. A. T. Thomson on Ioduret and Hydriodate of Iron.

To theorize and speculate is a characteristic of talent and genius. Dr. Thomson has long distinguished himself by his practical observations in general, but especially by his labours in chemistry and pharmacy. The following extract will, we think, illustrate the remark with which we commenced this short article.

"In reflecting upon the solubility of the Ioduret of Iron; and the striking difference, in this respect, beween that compound and its components;* and, knowing the value of these separately, as medicinal agents, I inferred that the curative influence of both might be greatly enhanced by the facility which the compound affords of introducing them into the system, in the state of combination. Another consideration which led me to suspect that this combination of Iron and Iodine would prove a useful medicinal agent, was my knowledge of the powerful influence of Iron as a tonic, when administered in the state of a Protoxide; and I was aware that it exists in this state in the Hydriodate, into which the Ioduret changes when it is dissolved in water. I supposed that, if this active oxide thus combined with Hydriodic acid were taken into the circulation, a circumstance extremely probable, the Hydriodate, meeting with an alkali, would suffer decomposition, and thus the two compounds would be enabled to exert their separate influence on the system, in a form and under circumstances the best fitted to render them efficient.

Now, there is much reason for supposing that the Hydriodate is decomposed in the system, and that the Protoxide of Iron, and the Alkaline Hydriodate which result, exert their separate influence on the nervous and the vascular organs; the former operating as a tonic on the vital solid, and the latter as a powerful stimulant to the capillary system. Or, if we take another view of the subject, keeping in recollection the striking influence of Chlorine on the solution of the Hydriodate out of the body, and knowing, also, that this agent is evolved in the course of the circulation, it is possible that the Protoxide of Iron, which is set free by the decomposition of the Hydriodate in the system, may be converted into the Proto-muriate of Iron, whilst the Iodine is evolved in a free state; and thus the same tonic and stimulant influence would be as powerfully exerted as in the former case. If either of these theories of the manner in which the Hydriodate operates be admitted as correct, the class of diseases in which it is likely to prove useful at once present themselves; namely, those in which the capillary system requires to be stimulated and the tone of the habit to be maintained, or to be brought up to the healthy standard:—Scrophulous affections, Tabes Mesenterica, Chlorosis, incipient Scirrhus, Rickets, Amenorrhœa, Bronchocele, Atonic Dyspepsia, and all conditions of direct debility."

We have a very humble opinion of the amount of our knowledge as to the *modus agendi* of medicines in the interior of the body. The chemistry of the living alembic is to us a *terra incognita;* and therefore the ingenious theory of our author is as good as any other. It is of more importance to know that Dr. Thomson has successfully employed this combination of iron and iodine in the various forms of scrofula, of which forms he has adduced cases in illustration.

"As the Hydriodate of Iron possesses both the stimulant properties of the Iodine, and the tonic powers of the Protoxide of Iron, the probability that it might produce all the beneficial, without the deleterious, effects of iodine, was presumed; and I was, therefore, induced to order it in *scrofulous* affections. The results that have followed its administration have been such, that I have no hesitation in regarding it a most valuable addition to the means which we already possess of treating the *chronic* forms of these diseases."

---

* It is scarcely necessary to remark that metallic Iron is insoluble, and that Iodine requires seven thousand parts of water for its solution.

It is to be borne in mind, that the medicine in question is a stimulant; and that, when marks of irritation in the primæ viæ exist, care should be taken to clear the bowels, and remove, if possible, the causes of irritation, before exhibiting the remedy. The following form, we perceive, is that which our author generally employs :

℞. Ferri iodureti, gr. ij. ad gr. iv.

Aquæ distillat. ℥xj.

Tinct. aurantii, ℥j.

Ft. haustus ter die sumendus.

Cases are adduced by our author, and by respectable correspondents, where the remedy was found useful in chlorosis, in carcinomatous diseases, in syphilitic cachexiæ, &c. for which we must refer to the pamphlet itself. As each of the ingredients, singly, is of known efficacy in many diseases, we consider it very probable that the combination will be found a valuable formula.

---

A Dictionary of Terms employed by the French in Anatomy, Physiology, &c. &c. By Shirley Palmer, M.D. Part I. July, 1834.

This is an extraordinary work, and, if completed, will be a very valuable one. We learn from the advertisement, that—

" The present work has been undertaken with a view of facilitating the perusal of French and German literature to the Medical Student or Practitioner who appreciates, as they deserve, the literary productions continually emanating from the press, in France and Germany; and who feels honourably solicitous to appropriate to himself, from their original source, the valuable facts, and the novel and often important views and opinions with which these productions teem. It may also serve to refresh his recollection, ever fleeting and evanescent without practice, of the elements of the Greek and Latin languages.

" How far the work, now offered, is calculated to achieve such objects, experience can alone determine. It was not undertaken in a rash and unreflecting spirit: it has not been, thus far, executed without great labour and research. Had a longer period for its completion been allowed him, or had the Author been able, during such period, to concentrate his attention more exclusively upon the performance, the numerous errors and deficiencies, which the enlightened and experienced eye will readily detect, would not have been suffered to escape correction.

The *Second Part* will appear about December next,—the *Third* and last, with Title-page Preface, and an Index of the German terms, in the Spring of next year."

It is, as we said, an extraordinary work, full of erudition and pregnant with research. We cannot, of course, give an analysis of a dictionary; but, by exhibiting one or two specimens, the reader will be enabled to judge of the work.

" Anévrysme, Aneurysme, s. m. —*ἀνεύρυσμα* (*ἀνευρύνω*, I dilate)—aneurisma, aneurysma, n. L.—aneurisma, anevrysma, n., pulsadergeschwullst, f., die erweiterung einer arterie, G.—aneurism, swelling, dilatation of an artery. Aneurism may be defined, a tumor, formed by arterial blood from dilatation, rupture, or division, of the coats of an artery. The term has been also applied, by some writers, to dilatation of the cavities of the heart, and even to enlargement of the organ from thickening of its parietes.

Aneurism shews itself under three different forms: 1. that of *true* aneurism—*vrai*, F.—aneurysma *verum*, L. —das *wahre* anevrysma, G.—formed by dilatation, circumscribed or diffused, without breach, of all the coats of an artery. In the *former* case, it constitutes the variety called *circumscribed*—circonscrit—circumscriptum, umschreibene;—in the *latter*, the *diffused*—diffus—diffusum—ausgebreitete—of *true* aneurism: 2. *false* or *spurious*—*faux*—*spurium* — das *falsche* anevrysma — formed by a breach of two or all of the arterial tunics, and presenting two varieties;—the *circumscribed*, in which the blood, escaping through a rupture of the internal and middle coats, converts the external coat of the vessel into an aneurismal sac;—and the *diffused*—where the external coat, also, has subsequently given way, and the blood been poured out into the surrounding cellular structure: 3. *mixed* aneurism—*mixte*—*mistum*—das *gemischte*—which likewise comprehends two varieties; one, the *internal,* consisting of an hernia-like protrusion of the internal, through a wound or rupture of the middle and external coats of an artery;—and the other, *external,* produced by rupture of the dilated coats of true aneurism, and consequent diffusion of its contents through the circumjacent membrane.

Besides these principal forms, there is *Aneurism by Anastomosis*—anévrysme par anastomose, F.—das anastomotische anevrysma, G.—apparently caused by aneurismal dilatation of the extreme vessels of a part, and extravasation of blood into the distended cells of the cellular structure.

*Aneurismal Varix*—Varice anevrysmale, F.—das anevrysmatische Venengeschwulst, G.—is said to exist, when, from the transfixion of a vein, and penetration of the subjacent artery, by a lancet, or other sharp instrument, and consequent adhesion of the two vessels, a direct communication has been established between them; and the blood, flowing from the artery into the vein, dilates the coats of the latter into a sac. If, however, from the obliquity of the wound or other circumstance, such communication be not direct, but take place through the medium of an aneurismal sac formed by dilatation of the wounded artery, and interposed between the vessels, the disease is termed *Varicose Aneurism* — variqueux, F. — aneurysma varicosum, L.—das varikose anevrysma, G.

Aneurisms, from their situation, are, lastly, distinguished into *internal* and *external.* To the *former,* belong aneurisms of the cerebral, and especially of the basilar, arteries—probably a frequent source of fatal apoplexy—and of the thoracic and abdominal aorta: to the *latter,* aneurisms of the temporal and carotid arteries, and of the larger arterial trunks of the extremities. For a minute description of the varieties, formation, and distinguishing characters, of the disease, consult Hodgson's *Treatise;* and Art. *Aneurism,* in Cooper's *Surgical Dictionary.*"

"CLIMACTÉRIQUE, adj.—climactericus (κλιμακτὴρ, every seventh year of human life), L.—klimacterisch, G. The ancients believed that human diseases were developed with greater frequency and fatality in certain years than others: and, hence, every seventh year was called by them, the *climacteric* year—*année climactérique,* F.—κλιμακτηρικὸς ἐνιαυτὸς—annus *climactericus,* L.—stufenjahr, n. G: while the sixty-third, as a multiple of 7 by 9, and therefore peculiarly pregnant with mortal ailments, was distinguished by the title of the *grand Climacteric.* Some physicians have also termed *climacteric*—*époques climactériques,* F.—certain periods of life, as characterized by revolutions in the human economy not dependent on the numerical progression of years. Such are the period of puberty in both sexes; and that of the cessation of the menstrual flux, in the female."

We sincerely hope Dr. Palmer will complete the work, which will form a monument to his industry—an extremely useful Dictionary for all who consult the works of our Continental writers.

---

### MR. GUTHRIE'S OPERATION OF TYING THE COMMON ILIAC ARTERY.

In the 39th number of this Journal, we mentioned that Mr. Guthrie had tied the right common iliac artery for what appeared and was deemed an aneurysm. The operation was thought to be successful, but the patient has since died, and the truth of the Hippocratic axiom—observatio difficilis, experientia fallax—has been illustrated by the event.

The Medical Gazette contains a long and an interesting lecture delivered by Mr. Guthrie on the subject. Much is needlessly apologetic, for Mr. Guthrie can afford to be mistaken. When we subject the lecture to a gentle pressure, its solid residuum is this.

The patient, a lady, was not young, yet amidst the abundance of statements and of information, we find no mention of her age. She had laboured for some time under pain in the hip, when she accidentally struck it. Soon after this a tumor appeared, and when it had attained the size of her fist, it began to beat " like her heart." It continued to increase, and after the lapse of a year she came to town. At this time, the tumor was as large as an adult head, situated on the right buttock, and so inconvenient as to prevent her from lying on that side, or even on her back. Separate examinations were made by Mr. Guthrie, Sir A. Cooper, Mr. Thomas, and Mr. Keate. On the whole, they concluded that the tumor was aneurism, and the common iliac was tied by Mr. Guthrie.

We believe we must be indulged with Mr. Guthrie's own account of the operation.

" I began the operation by placing her on a table, as much on her back as the tumor would allow, and by making an incision upon the fore part of the belly extending, beginning below the inside of the anterior spine of the ilium. about an inch, carrying it upwards and diagonally inwards towards the edge of the rectus muscle above the umbilicus, so that the incision was between six and seven inches long. I may state, that if the incision is made in the side, from the ribs to the ilium, in a straight line, the greatest possible difficulty is experienced in turning over the peritoneum so as to place your finger upon the last vertebra; but if a diagonal inclination be given towards the rectus muscle, not opening its sheath so as to expose it, but carrying the incision fully np to that part, then there is room to turn over the peritoneum with its contents, so as to get at the artery.

After dividing, then, the skin and the common integuments, the three muscles were of course also divided in layers; the division of the latter, the transversalis, was attended with very considerable difficulty, inasmuch as there was little fascia transversalis, and the peritoneum was remarkably thin—as thin as the common white silver paper, or nearly so, that is used for ordinary purposes. On attempting to reach the under part, on the inside of the ilium, so as to push the peritoneum over, I found this could not be done; that the tumor had extended inwards; and some bleeding took place from the large veins which surrounded it, giving rise to the caution not to proceed farther in that direction. At this moment, in spite of the greatest possible care that could be taken by Mr. Keate, who protected the peritoneum, a little nick took place in it, and the small intestine made its appearance below. I then tried to gain a greater extent of room upwards; but where the tendon of the transversalis passes directly across to form the sheath from the lower ribs, the peritoneum is usually so exceedingly thin, and so closely attached to it, that it can scarcely be separated but with the greatest difficulty. I knew this from an operation of a similar kind which I performed at the Westminster Hospital the year before, and in spite of all the precaution that I could then take, the peritoneum was on that occasion injured. I was so well aware of the great probability of its being wounded at this part, that I took double precaution; but in spite of all the care that could be taken, this part of the peritoneum was opened, and the right lobe of the liver made its appearance through it. The opening on the fore part of the belly was not large enough to admit the two hands, and there were two openings in the peritoneum, one above, and the other below, each of them much disposed to increase in size by the moving of the patient; and the operation did not seem to be the most agreeable one that could be performed. The peritoneum, however, being separated a little from the posterior wall of the abdomen from the outside, four fingers of one hand could now be introduced under it, and turned a little over towards the opposite side.

There is a point here of great importance to recollect, and it is, that the peritoneum must be raised over without the hand being pushed back towards the posterior wall of the abdomen but as little as can be avoided; for there is some fat usually at that part, if there be any to be found in the body, and behind which you are very apt to get in performing the operation, instead of going in the front; and if you do, it leads to the under edge of the psoas muscle instead of the upper, and renders the operation much more difficult.

The peritoneum being now separated from behind, and carefully turned over, I found I could only get one hand, or a little more, underneath in search of the artery, and that no more room could be obtained by increasing the incision upon the fore part of the belly. Under these circumstances it became obvious that, to seek in the dark, without being able to have the advantage of sight, for the internal iliac artery, which can hardly be found at any time but with difficulty, was not likely to be attended with success, and I therefore determined upon placing the ligature on the common trunk of the artery. In order to effect this, I separated the peritoneum, passed my finger across the psoas muscle until it rested upon the fifth lumbar vertebra; and I now thought, of course, that I must feel the common iliac artery; it was not, however, to be felt. I passed my finger up as high as the fourth lumbar vertebra, trusting that I should feel the end of the aorta; but even that could not be felt, from a circumstance that I was satisfied had occurred in the previous operation, which is not known in surgery, but which I will now state: it is, that the common iliac artery rises with the peritoneum, which I believe the vein does not. My finger then, resting upon the spine, was beneath the vessel I was searching for. Mr. Keate endeavoured to raise the peritoneum, to give me an opportunity, if possible, of seeing the vessel; but that was quite out of the question; the incision, however long, was not sufficient to allow this to be done. However, on raising the peritoneum a little, he felt the pulsation of the external iliac artery; and I now then, passing my finger upwards, found the common iliac adhering to the peritoneum. I separated it carefully with the point of the fore-finger of the right hand, with the finger and thumb of the left—for no more room could be given—I passed the aneurismal needle, and placed a common thread ligature (now on the table) round the artery, which was done without seeing it. In an operation like this, you must have your eyes at the extremities of the fingers, and your head in your hand. I could bring the artery a little forward, by means of the aneurismal needle which was underneath it; and in this manner it was brought into view. It appeared to be perfectly clear; and I calculated, from feeling the division of the aorta above, that the artery was tied exactly in the middle."

We need not pursue closely the subsequent details. On the following day the patient required bleeding, and was bled. The temperature of the limb was preserved by constant frictions, with the application of hot bottles to the feet.* The operation was performed on the 24th of August, and on the 19th of September, the ligature came away. The tumor rapidly diminished in size, and, in a month, it lessened by one-half. In the course of two months the wound had healed, with the trifling exception of one small point. In December or January the patient went to Scotland. The tumor again augmented in size, and on the 30th of April the patient died, exhausted by disease.

Perhaps it is not necessary to state more than that the tumor was not an aneurism, but a malignant growth, of the nature of medullary sarcoma. The common iliac had been tied about its middle, where its cavity was found, on dissection, to be obliterated. It was pervious above and below, to an eighth of an inch from the actual site of the ligature. No coagulum had consequently formed between the latter and the nearest branch arising from the ves-

* De Graefe, who saw the patient, recommended the application of cold water to the limb, to maintain vital heat! How strange that the ablest foreigner has always an absurdity to set off against his merits. His organ of common sense is seldom free from a flaw.

sel, or from the trunk of which it was a continuation. The internal iliac on this side was enlarged—the external iliac was unaltered—the principal channels of the collateral circulation were the epigastric and the circumflexa ilii.

The case is one of great interest, of some importance. It does honour to Mr. Guthrie as an operative surgeon, and no discredit as a practical man. We may venture a few remarks on the details.

It is evident that a mistake was committed in the diagnosis. Yet the character and the experience of the Gentlemen who made it affords a guarantee to all who are possessed of candour and judgment, that the error was more owing to the imperfection of science, than the fault of the individuals. It is a fact of which many members of the profession are, perhaps, not sufficiently aware, that very great difficulty frequently exists in distinguishing pulsating medullary tumors from aneurismal sacs. The ordinary means of diagnosis applied to tumors receiving a pulsation from arteries above, beside, or beneath them, are inapplicable to pulsating fungoid tumors, for the obvious reason, that their pulsation is occasioned by their own blood-vessels. It might seem that the capability of emptying a sac by pressure would enable the surgeon to distinguish it from a tumor not containing fluid, and therefore not admitting of diminution by evacuation of its contents. But in some cases of aneurism, the deposition of lamellated coagulum is such that pressure effects little alteration of their size: and pulsating malignant tumors contain so numerous or capacious blood-vessels, that pressure may exert a considerable influence. We need not pursue these considerations farther. It is certain that the diagnosis between the two diseases is occasionally so obscure, that the most experienced and judicious surgeons are liable to error, and have committed a mistake. We may allude to an instance which we witnessed a few years ago.

The patient was a lady, from forty to fifty years of age. The tumor was situated beneath the crest of the right ilium. It extended somewhat upwards into the abdomen, outwards over the natis, downwards on the thigh, and inwards to about the centre of Poupart's ligament. Some large veins meandered on its surface. It pulsated synchronously with the heart. A large artery was felt entering it anteriorly, and another was distinguished behind, in the situation of the sciatic branches. Pressure on the common femoral artery was said to arrest the pulsation in the tumor. The tumor, when first seen by the surgeon, (an experienced and a good one,) whose patient the lady was, had little or no appreciable pulsation. The surgeon considered it an abscess. The tumor began to pulsate, and the surgeon then thought it fungus hæmatodes. In order to decide the matter, he punctured the tumor in two places with a grooved needle, when there issued from each puncture a *pulsating* jet of arterial blood. This circumstance appeared so decisive of aneurism, both to him and to other surgeons who saw the case in consultation, that the common femoral artery was tied. The ligature on this vessel had not the effect of commanding the pulsation in the tumor. In six days after the performance of the operation the patient died. The pulsation in the tumor had never ceased. The tumor, on dissection, was highly vascular, and of medullary consistence. The external circumflex artery passed into it.

We have heard of another case, in which a mistake of this description was committed. The publication of Mr. Guthrie's case will have the good effect of directing attention to the subject, and though it must be owned that it does not enable us to fix on any symptom as peculiarly characteristic of either disease, it is calculated to teach the surgeon to pause, and to hesitate more than he has previously done in performing a serious operation for the cure of a pulsating tumor. Caution in such cases is now more indispensable than ever, and perhaps this alone may prevent the frequent occurrence of mistakes. In the present state of science, it is probable that such occurrences are more likely to be avoided by attention to the history of the complaint, to its seat, its general features, than by resting on any particular character. The "purring thrill," for instance, or the "whizzing" said to be diagnostic of aneurism, are now well known to be produced by pressure on any artery of large dimensions.

A word or two may be permitted on the operation itself. To say that it redounds to the surgical reputation of Mr. Guthrie, would be merely to express, in imperfect language, the feeling which all must entertain. We may add two remarks of that gentleman upon the subject.

"It was not formidable from the bleeding, but from the circumstance, that room could not be got for the introduction of the two hands; and, strange as it may appear, the whole safety and ease in doing the operation consist in the incision being so large at the fore part of the belly, that the bowels and the peritoneum may be freely turned over by the two expanded hands, so that you can see what is going on underneath. In the former case, the whole parts could be seen in the peritoneum distinctly, and several gentlemen not in the profession saw the iliac artery in its natural situation. I have no hesitation in saying, that, in the manner I have described, the operation of applying a ligature on the aorta might be accomplished without the least difficulty. I could as easily have tied the aorta as the common iliac artery; it was only necessary to have gone an inch higher. If at any future period it is necessary to pass a ligature round the aorta, it is in this way the operation should be performed.

But this operation will lead to another important result; I believe it will prove that it will never be necessary again to put a ligature upon the aorta. It has been twice attempted, and in both cases the patients lost their lives, just as they would have done if the operation had not been performed. If you examine the parts in the dead body, you will find that, if you can only apply a ligature three-quarters of an inch from the termination of the iliac on one side, that it requires little knowledge to pass one over the iliac artery of the opposite side; so that, when an aneurism has been formed, and you cannot tie the iliac artery of the side in which the disease is situated, you may be enabled to do it in the way I did this operation—from the opposite side, and it is not rendered necessary to tie the great trunk of the aorta at all. That is one of the most important results which I think may be deduced from the operation I have described."

Mr. Guthrie indulges in an observation on the consequences of the application of the ligature to the artery, to which we shall venture to allude.

"A little opening was made in the artery above and below where the ligature was tied, in order to observe the state of the vessel; and it appeared that it was pervious to within the eighth of an inch on each side of it; so that the ligature just cut the artery through, and the two cut edges united by adhesive inflammation above and below, without forming a coagulum, and without diminishing up to the next collateral branch—a fact which I have said always existed—but which is quite contrary to the received opinions of the present day."

If Mr. Guthrie intends to affirm that coagulum is never deposited in an artery, between the part tied and the nearest branch, there are numerous well-attested facts that might be mentioned, which establish the occurrence. Surgeons have also been aware, that at times no coagulum of this sort is formed, and yet that secondary hæmorrhage does not follow. We presume that the fair interpretation of the passage is, that Mr. Guthrie has long been inclined to deny the necessity, and disbelieve the frequency, of the deposition of such a coagulum. The present case is remarkably in point.

Before we quit the case, we must venture to observe, that we cannot understand how the common iliac artery could be carried forwards with the peritoneum. Its anatomical relations would seem, *à priori*, to forbid such a displacement.

Again we offer Mr. Guthrie our congratulations on the manner in which he performed the operation. Much as it does him honour, his candid narration of the issue of the case is calculated to increase his genuine reputation.

---

Reclamation of Valentine Mott, Esq. with Reference to the Operation of Tying the Common Iliac Artery.

We cordially publish the following letter from Mr. Valentine Mott, one whose reputation should be dear to America. It is singular that it should arrive at a time, when the result of the operation performed by Mr. Guthrie on the common iliac artery has been such, as to give the palm of success to Mr. Mott. But success is, after all, a false touchstone of merit; and the character, both of Mr. Mott and Mr. Guthrie, will be measured by a fairer and a higher standard.

*New York*, 25, *Park Place*, *June* 10*th*, 1834.

Sir,—As the individual upon whom I operated, in the year 1827, for ligature of the *arteria iliaca communis*, is still living, may I ask of you the correction of the following sentence, in the "Medico-Chirurgical Review" for Jan. 1834. "Thus this most formidable operation has been successfully performed *for the first time*, and while it adds a wreath of laurel to the brows of the distinguished surgeon, it exhibits a splendid triumph of British surgery."*

It is worthy of remark, that Mr. Guthrie has committed the same error in his work, p. 365—" on the Diseases and Injuries of Arteries," especially as the case is quoted from the " American Journal of the Medical Sciences," the last paragraph of which is in the following language :—" The gratification his visit afforded me is not to be imagined, save by those who have been placed under similar circumstances.† The *perfect success* of so important and novel an operation, with the *entire restoration* of the *patient's health*, was a rich reward for the anxiety I experienced in the case, and, in a measure, compensated for the unexpected failure of my operation on the *arteria innominata*."

While justice to American surgery requires that I should make this statement, I shall always be ready to award to the British school the merit it is so justly entitled to, and of which I am proud of acknowledging myself to have been a pupil.

With great respect, I have the honour to be,
Your obedient Servant,
Valentine Mott.

*To* James Johnson, M.D.
*Physician Extraordinary to the King.*

* Mr. Guthrie's case.

† The patient having come twenty-five miles, nearly three months after the operation.

---

Fracture of the Skull, with Depression. Occurrence of Convulsions, and Cure.

Mr. Cotton, an intelligent surgeon of Bagshot, has communicated an interesting case of this description to the Lancet.* We shall briefly state the particulars.

*Case.* A boy, æt. 14, was thrown against a gate, and trodden by a horse, at 10, a.m. of the 4th July. There was a lacerated scalp-wound above the external canthus of the left orbit—a puffy swelling on the zygomatic arch—and a simple fracture of the parietal bone, embracing its posterior half, and extending obliquely forwards, to the junction of the squamous portion of the temporal with the greater ala of the sphenoid bone. The bone was depressed, and the scalp much puffed. There was bleeding from the nose and mouth—there was a state of drowsiness, alternating with shrieking, and

* Aug. 9th, 1834.

efforts to remove himself from the bye-standers—the pulse was labouring and quick.* The head was shaved, and cold lotion was applied, shortly after which the patient was seized with violent convulsions, and distressing attempts at vomiting. These symptoms continued for an hour and a half, and ended in complete insensibility. The pulse was now labouring, irregular, 56—the pupils permanently dilated, and insensible to the application of a lighted candle—the respiration stertorous.

*VS. ad ℥xxx. ℈j. of Calomel, placed upon the fauces. Enema of muriate of soda, colocynth, and jalap.*

Several copious evacuations were procured, and at 2, p.m. four hours after the accident, the symptoms were somewhat relieved. At 7, p.m. the patient was sensible on being roused, and complained of excruciating pain in his head. Pulse soft, 76—skin warm—tongue furred. The slightest pressure on the seat of fracture produced convulsive movements. *Lemonade was ordered as a drink, and salines with Epsom salts were prescribed as medicine.*

He passed a restless night, repeatedly attempting to get out of bed, and talking incoherently. Next morning, the skin was hot and dry—the pulse sharp and 86—the tongue furred—the pupils sluggish and dilated. He was drowsy, but conscious when moved, and still complained of severe headache.

*VS. ad ℥x. Calomelanos, gr. ij. o. 3tiâ horâ. P. c. aliis.*

In the evening, the symptoms were sensibly relieved, and after that time, the amendment of the patient was progressive, though slow. We need not pursue the details. The report of Mr. Cotton concludes upon the 21st, seventeen days from the reception of the injury. The wound of the scalp was then healed. No unpleasant symptom remained, excepting a slight deafness. There was no perceptible depression of bone, "but an evident callosity in the course of the fracture."

Whatever the nature of the fracture and extent of the depression may have been, a careful consideration of the particulars of the case will convince the experienced surgeon, that moderate pressure on the brain existed. That the pressure *was* moderate, is proved by the presence of convulsion, and concomitant absence of long-continued stertor and coma. The observations of Mr. Brodie, the records of facts, and the study of the symptoms and pathology of hydrocephalus, establish the important fact, that convulsions are a consequence of comparatively slight compression of the brain.

The treatment of the case before us was as judicious as successful. We may equally approve the early and the secondary management—the application of cold, the venæsection, and the purging in the first instance—the steady exhibition of calomel, when the symptoms of fever and the pain in the head became indicative of the supervention of inflammatory action. This is the practice we have taken repeated opportunities to recommend, and it gives us great pleasure to offer Mr. Cotton the advantage, small as it may be, of the cordial approbation of this Journal.

---

### THE CHOLERA.

Our readers may probably remember that, when the cholera prevailed, we devoted much space and some trouble to the subject, and endeavoured to lay before the medical public all that was interesting, important, or novel with respect to it. We see no reason to regret the course we thought it prudent and right to pursue. We endeavoured to allay unnecessary apprehensions, and dispel unreasonable terror—we denied the virulent contagiousness of the malady, and doubted its power of extensive mischief in this country—we denounced vexatious quarantine regulations as useless, and, consequently, bad—we reprobated the attempt to coerce the dearest feelings and the prejudices of the people by legal enactments and the interference of police—we ridiculed the mummery of fasts and

* It seems difficult to reconcile the two conditions.—REV.

fumigations, and midnight burials—and last, not least, we did our best to expose the enthusiasm, or the quackery, of "legally qualified" gentlemen, who confidently published their nostrums and their cures, at a time when the mortality was notoriously at its highest.

We do not intend to re-argue the nature and the causes of cholera. Of the former, we know as little as we ever did—and the latter are enveloped in equal obscurity. Extreme opinions have, we trust, been softened down—the bitterness of party has expired with the occasion—and we think we may assert, that the majority of well-educated, practical, and reasonable men have arrived at nearly the same conclusions, and act on very similar principles. None dream at present of advocating quarantine—few entertain the idea or the wish of alarming the public with the notion of contagion—all are convinced that, in the great mass of cases, care on the part of the individual affected, and judgment on that of the medical attendant, will be amply sufficient to obviate the occurrence of malignant symptoms, or of serious consequences—and most are convinced, also, that when, from imprudence, from inattention, or from any other cause, the worst features of the malady have appeared, remedies of all descriptions are too generally powerless. The common sense of intelligent practitioners has led them to regard the vaunts of successful treatment, in *such* cases, as idle fancies or unblushing falsehoods.

Those who are familiar with the history of cholera are probably aware, that at the time when it was committing its greatest ravages in Hindostan, the newspapers of that province teemed with the accounts of successful remedies. So fatal were the consequences of these daily cures, that the Government was compelled to prohibit their publication. We do not know that the evil has been quite so formidable in this country; but, whenever the disease has been most prevalent and fatal, we have always remarked that most specifics, or pseudo-specifics, are made public. In the Autumn of 1832, the weekly, the monthly, and the quarterly medical journals were rife with proposals of the most opposite descriptions, resembling each other only in their success. Mr. A. amazed the public with a series of cases of the most malignant cholera, triumphantly treated with calomel and opium—Mr. B. looked with horror on that murderous practice, and effected all his cures with cold water—Mr. C. was successful, by preventing his patients from slaking their agonizing thirst, although, like Dives, they implored but one drop to wet their lips. Dr. D. administered fluids by bucketsful through the mouth, the anus, and the veins. All were sanguine, all victorious, and the difficulty was, not to discover a specific, but to choose one. Some simple persons were delighted with all this, and rapturously received each Shiloh as he came. But the rapid succession of miracles and prophets at length proved too much for even the most credulous digestion. The true believers were forced to confess that they had thrown away their faith on false divinities, and were compelled to fly for comfort to the fond idea, that a genuine specific existed somewhere, though, like Solomon's seal, its possessor was unknown. It is not impossible, said the Lancet of that day, "that a specific for cholera has already been discovered."

It may seem that we are talking of things that were, of follies irrevocably past, and of credulity not likely to revive. A moderate acquaintance with mankind is sufficient to stamp the truth of the remark, that experience seldom instructs the mass. The deceived and abandoned credulity of yesterday is enacted on the stage of to-day, and the same performers are often seen to play again their proper parts. His Majesty's medical subjects are, at this very moment, exhibiting a pantomime of this description.

When the epidemic cholera first commenced its career in this country, the practice in which most confidence was placed was venesection, and the exhibition of calomel and stimulants. A brief experience was sufficient to convince all unprejudiced practitioners, that cases of a really formidable character were not amenable to these remedial measures, however rational their employment might appear. Then succeeded, at a rapid pace, the advocacy and the trial of innumerable modes of treatment, some more and some less plausibly supported, but all, without exception, brought forward by their parents as successful, in cases of the worst description. It would be tedious, and it is unnecessary, to recount in due order the rise and fall of these successive methods; but a brief enumeration of the principal may be of service. Waiving a very strict attention to chronology, they may constitute the following list:—Stimulants and frictions—large doses of calomel —small doses of calomel—calomel and opium—emetics of mustard, of common salt, and of tartarized antimony —salines by the mouth, by the anus, by the veins—water of all temperatures and in all quantities—no water at all—the application of heat—the application of cold—drugs beyond number, and of every conceivable mode of operation—bandages on the abdomen. We will not fatigue our readers nor ourselves by attempting to extend this heterogeneous catalogue. But one observation may be usefully permitted. Each successive contributor of a method or a drug denounced the mischiefs, or deplored the inertness, of those which had already been proposed. The water doctor cried "murder" when calomel and opium were employed —the patron of emetics pitied the weakness of the water doctor—and the sturdy advocate of calomel or of stimulants looked with derision upon both.* Amongst the audience that surveyed the performance of this play of many parts, some individuals looked on with philosophical incredulity and apathy; but the multitude were caught by each clever juggle, and rapturously cheered each dexterous actor.

From the hasty sketch of what *has* occurred, let us turn for a brief space to the present scene.

Early in the Autumn, many cases of diarrhœa, and some of cholera, were observed in the metropolis. Towards the latter end of August, a sudden and a great alteration took place in the temperature and in the weather. The thermometer sank rapidly fifteen or twenty degrees, and sultry days were followed by nights of actual coldness. The attacks of diarrhœa multiplied, and insulated instances of malignant cholera soon became whispered abroad. Few medical men, in extensive practice, failed to see decided cases of the malady, and, about the close of August, its prevalence and its severity had become incontestable. Now came the private panic and the public gossip—dietetic *leaders* in the weekly Journals —and a dinner of the market-gardeners, at which fruit was ostentatiously devoured, and cholera boastingly defied —and now, above all, came the insulated cure or the general specific—the drama, in short (a tragedy and farce), of the previous years was advertised again, the old dresses furbished, and the old actors rouged.

It seemed as if the fond illusion of the Lancet was realized at last—as if the specific which it thought might exist, but had not been discovered, was at length presented to the public. In the Number of that Journal for the 23d of August, a communication appeared from Mr. Beaman, announcing a remedy "so extremely successful," that he laid it before the profession and the public. That remedy is applicable, and Mr. Beaman desires that it may be thought so, to cholera, in its worst and most aggravated form. In that form, it had cured "no less than eleven consecutive cases." It must be owned, that a remedy which can lead to a happy issue so great a number of malignant cases must, indeed, be a boon to afflicted humanity. It is—emetics of common salt!

Many might deem that the plan of

---

* This is no idle flourish of words. Every observation in this article, sarcastic or rhetorical as it may appear, is founded on the recollection of circumstances that have occurred.

emetics had already been sufficiently tried and found wanting; and that the melancholy experience of preceding years had demonstrated their real value. Our contemporary does not think so. He considers the communication of Mr. Beaman as "one of the most important that has been made connected with the treatment of malignant cholera"—he entreats his brethren to try the remedy, and to note down the results of their observations—and he opens an editorial cholera-box, not "for long detailed cases, which would require much time in the *concoction,*" but simply for those leading facts sufficient to answer every good public purpose. In a subsequent number a mild attempt was made by another gentleman to urge the claims to public notice of the "pil. ceræ phos."* But the editor checked the impertinent intrusion, and observed that the preference must be given to communications illustrative of the effects of salt-water emetics. Mr. Wardrop, perusing with attentive satisfaction the important observations of Mr. Beaman, forwarded without delay some remarks from Dr. Hoskins, a communicant from Guernsey. Those remarks are so brief that we will venture to insert them.

"When the disease visited us two years ago, we found common salt and water the best remedy. Its superiority to any other plan forced itself upon our attention, and our Cholera Hospital became extremely popular as a medical asylum, with all ranks and classes. I may mention, that out of sixty-two cases, admitted into the hospital in the third stage, and treated solely with solutions of salt, forty-six recovered, and only sixteen died, whereas the mortality following the opiate and stimulant treatment was about sixteen in thirty-seven. At first I adopted the opiate plan, but soon afterwards became a convert to the saline."

Mr. Wardrop and the Lancet would appear, unless we misconceive the words and the meaning of Dr. Hoskins, to have misunderstood his treatment. He makes no mention of salt and water as an emetic, but gives it after the *saline* fashion. This is a different affair.

It requires no gift of prophecy to foretel, that before this article is published, salt and water emetics will probably have given way to some other equally successful plan of treatment. We would not swear that the pil. ceræ phos. will not be in vogue on the first of September, and we think it very likely that the immortal Dr. Stevens will be actively dispensing the inestimable blessings of salines. Damaged rice, we perceive, is pouring into the market, and since Dr. Tytler, Cassandra-like, is unheeded, there seems every reason to believe that the next few weeks will be prolific in opportunities for the friends of humanity to perform their cures and to publish their specifics.

In conclusion, we may offer a few observations on what appears to us the most rational manner of regarding and of treating this singular complaint.

The variety of symptoms displayed by different individuals would seem to preclude the probability of any single remedy becoming applicable to all. If we take the worst cases, we find in one the vomiting distressing and incessant —in another, purging is the prominent characteristic—in a third, the collapse is out of all proportion to either of the former symptoms. In this, as in every other disease, the practitioner should study the features of each case, and regulate his practice by the character they wear; he should also consider the age, the constitution, the habits, and the powers of the patient. In the majority of instances, diarrhœa continues, as before, the preliminary symptom. We have heard it remarked, that collapse is more sudden, and death more rapid than in former years. Our own observation would lead us to doubt the correctness of the supposition.

We have never found the diarrhœa

---

* This useful abbreviation is probably typical of pilula ceræ phosphorata, a compound, we may suppose, of phosphorus and wax. We presume that this mixture of combustible materials is chiefly adapted for the cold stage.

resist a dose or two of calomel or blue-pill, combined with opium and some essential oil, and succeeded by aromatic draughts containing rhubarb with hyosciamus, and generally the liquor opii sedativus. These remedies, assisted by quiet, confinement, and restriction to slops, have not failed in any instance we have seen to arrest an attack of diarrhœa.

In cases of cholera, that is of vomiting and purging, of moderate severity, the exhibition of small doses of calomel and opium, accompanied with effervescing draughts containing laudanum, and with opiate enemata, if the purging is considerable, are the means we usually employ. The patients should, of course, be confined to bed, and warmth or mustard sinapisms must be used, if necessary. Emetics are always beneficial in the first instance. They sometimes arrest the vomiting immediately, and they always appear to facilitate recovery. Beyond this we doubt if emetics are of much service, and certainly we would not dream of allowing them to usurp the office and the place of the other remedies we have mentioned.

In the very worst cases of cholera we really believe that it signifies little what may be done. Death occurs in the great majority, and those that have done well, have been treated in such various ways, that it appears impossible to attribute much to any one method. Under such circumstances, the safest practice is usually to trust to those general principles, which are themselves the sum of many facts, and the results of the extensive observation of disease. If much coldness and collapse are present, the application of external heat and of sinapisms appears reasonable and has proved of service—if the thirst is excessive, fluid should be granted, the quantity and the temperature being regulated by the wants and the wishes of the patient—if the vomiting is very violent and continual, the less that is given by the mouth the better—the purging should be met by starch injections with opium—and if prostration is urgent, stimulants in some shape do really appear to be imperatively required. We must say we are advocates for the moderate employment of calomel. If the vomiting and purging are severe, it is better to combine it with opium—if not, the quantity of the latter should be small, or it may probably be usefully dispensed with. We have said that if sickness is a prominent symptom, it is better to avoid, as much as possible, exhibiting medicine by the stomach. Under such circumstances, when collapse was urgent, we have, on several occasions, adopted with much apparent advantage, the plan of administering every hour or two, enemata of small quantities of beef-tea, arrow-root, brandy, and laudanum in conjunction. In some cases of seemingly hopeless collapse, reaction was established under this management. Calomel, with or without opium — effervescing opiated draughts, if the stomach will at all retain them—enemata with laudanum if the purging is severe; of beef-tea, arrow-root, laudanum, and brandy, if sickness constitutes the principal feature—warmth and sinapisms on the surface—and the fluid the patient may prefer, commonly cold water, form our general system of treatment in the worst cases of cholera. There are many particulars on which we will not enter, and much must, of course, be left to the tact and the judgment of the attendant on the case.

Of bleeding we will only say that, although it is of service in robust individuals, and in the earlier stage of the complaint, it is seldom advisable under other circumstances.

But the surgeon or physician must carefully watch the symptoms that occur on the subsidence of the collapse. Then is the period for local congestion—then is the time when the seeds of irremediable mischief are sown in the head or in the intestinal mucous membrane. It must not be forgotten that those who die in the second stage, are generally cut off by inflammation or ulceration in the mucous membrane of the bowels: or, by congestion, perhaps inflammation of the brain. The case is one of typhus, in the true acceptation of the term; as such it must usually be treated. The moment any organ appears involved, leeches should be instantly applied and repeated as necessity requires. Calomel or hydrargyrus cum creta, in two or three grain doses, should be given three or four times daily—aperients of rhubarb to carry off depiaved, and promote the restoration of natural secretions, should be cautiously but steadily employed. Under these or similar measures many patients will recover, (we mean they have done so,) whose situation appeared most gloomy, if not actually desperate.

Such are the results of the experience of those whose opportunities entitle them to the privilege of pronouncing an opinion—who have looked at the disease as practical men—who have tried and witnessed various methods—who have no exclusive partialities, nor any object to attain—and who merely relate, in a hurried and imperfect manner, what they have found to be consistent with facts. Without attempting to discourage experiments, they cannot avoid expressing the belief that hitherto they have succeeded in establishing only the general inefficiency of any particular class of remedies.

---

### Case of Chronic Enlargement of the Clitoris, removed by means of Ligature. By James Edward, Surgeon, Forfar.

The following case has been communicated to us by Mr. Edward. He appears to have treated it with skill, and to have removed the disease with decision. We have seen several instances of similar alteration of the prepuce, of the clitoris, of the labia, and of the nymphæ. We think that excision is usually preferable to the employment of the ligature. It is more expeditious, less painful, and productive of no risk or inconvenience.

"In December, 1833, Mrs. Lindsay, about 40 years of age, consulted me regarding her complaints. On inspection, the clitoris was found to be about eight inches long, and of a pyriform shape. The pedicle of the tumor was firm, and about the thickness of the wrist; the most depending part of it hard, and fully larger than two fists. The nymphæ were elongated, and covered with a dry, smooth, and pale-coloured cuticle, pretty thickly set with small warts. The clitoris presented a similar appearance, except having none of the warty excrescences. The mucous membrane, having lost its secreting power, was become smooth and dry, and, by reason of the external position of the parts, was converted into an opaque, insensible cuticle. The sensibility of the parts, when elongated so as to project beyond the labia, was greatly impaired. With the exception, however, of being of a solid and fibrous structure, instead of a loose and spongy texture, they were not, in any other respect, morbidly changed. The disease was of two years' standing, and had commenced shortly after the patient's having undergone a course of mercury for syphilis.

While the external parts were held aside by an assistant, the clitoris was pulled out as far as possible from under the pubes, and a ligature applied close to the base of the tumor.

Excruciating pain was complained of during the first day, after which it gradually subsided. The ligature was tied every day for eight days, at the end of which the tumor dropped off."

---

### Literature of the Quarter.

We purpose, in this manner, to introduce to our readers a few works, which we cannot, with advantage, consider in separate and ceremonious articles. Our bibliographical record is devoted to the enumeration of books which we receive — our review department to works which deserve analysis, or must submit to criticism—and this corner may serve for the reception of those which merit some, but are not adapted for a lengthened notice. Let us look upon our list.

I. Illustrations of the Effects of Poisons. By George Leith

ROUPELL, MD. The Plates from Original Drawings, by AND. MELVILLE M'WHINNIE, M.R.C.S. Part II. Eighteen Shillings.

The former part of this useful work was noticed in a former Number of this Journal. The part in question contained four illustrations—the present exhibits a similar number. The first represents the effects of concentrated sulphuric acid—the second those of a large dose of oxalic acid—the third displays the consequences of a large dose of corrosive sublimate—and the fourth the condition of the mucous membrane produced by alcohol. The first illustration is drawn from poisoning and the human stomach; the originals of the other three are the stomachs of dogs that had been subjected to experiments.

1. *Effect of Concentrated Sulphuric Acid.*

A boy, æt. 2½, drank inadvertently some strong sulphuric acid. Immediately after swallowing the poison, he fell on the floor; when raised, his tongue was swollen and protruded from his mouth, and he complained of acute pain at the epigastrium. Medical assistance was quickly procured. The carbonate of magnesia was given, which occasioned active effervescence in the mouth. Castile soap in solution was afterwards administered; vomiting then came on, and acid matters, partly composed of food, were rejected from the stomach, followed by a large quantity of black, grumous blood. Leeches were applied to the epigastrium. No re-action followed the depression, which resulted from the injury to the stomach and œsophagus. The skin was cold. The pulse scarcely perceptible. The little boy remained perfectly sensible and in a state of comparative tranquillity until his death, which took place within twelve hours after the accident.

*Dissection.* The external marks consisted in vesication and discoloration of the lips, and in a hard, brawny patch on the left cheek, about the size of a half-crown, and of the colour of parchment.

The tongue and inner part of the mouth were of a dirty grey colour.

" The inner lining of the œsophagus was puckered, dry, hardened and brittle; it was readily detached from the parts beneath and came off in small scale-like portions.

The coats of the stomach were thin and allowed the contents to be seen through them. When opened the whole of the mucous membrane was of a dark colour, apparently stained by a bloody fluid, four ounces of which were contained in the stomach.

The large end was in no respect altered in texture, but the whole circumference of the smaller end, about midway between the œsophagus and pylorus, was black, irregular, rough and thickened. The change which had taken place here was the destruction of the mucous membrane and the effusion of blood from the injured portion, some of which had coagulated, and adhered to the part acted on by the acid.

There was no perforation of the coats of the stomach in this instance, nor was there any inflammation of the peritoneum. Life was apparently destroyed by sympathy of the brain with the injury to the stomach and œsophagus."

Dr. Roupell observes that the yellow stain, so remarkable in cases of poisoning by nitric acid, was no where visible in this instance. The plate representing the interior of the stomach and œsophagus is probably correct, and certainly well executed; yet it is devoid of that pictorial exaggeration and gaudy colouring so frequent in plates of morbid anatomy.

2. *Experiment to shew the Effects of a large Dose of the Oxalic Acid.*

Two scruples of oxalic acid, dissolved in an ounce of water, were introduced through a glass tube into the stomach of a small dog, by an aperture in the œsophagus, which was subsequently tied, to prevent vomiting. In a few minutes the dog made urgent attempts at vomiting; these were interrupted by cries of distress, and renewed with very little intermission until its death, which resulted in half an hour from the administration of the poison. In this case the animal remained curled up in the basket into which it had been placed after the operation.

(Another dog, to which a drachm of the acid was given, exhibited nearly the same symptoms: vomiting came on in five minutes; in ten minutes the hind limbs were paralyzed, and death resulted in twenty minutes. Muscular contractility was nearly destroyed in this case; the heart scarcely contracted on the application of stimuli, though made immediately after death.)

*Dissection, 24 Hours after Death.*—The œsophagus was natural in its appearance.

The stomach was vascular externally, and the hour-glass contraction was present. "It contained about four ounces of a dark-coloured, thick tenacious fluid at the larger end, which had stained the mucous surface. The coats were generally pulpy, softened, and had a white transparent appearance, but no perforation had taken place.

The duodenum was red, and presented dark coloured lines corresponding with the projecting rugæ, which had the appearance of being charred."

Dr. Roupell remarks, with apparent justice, that the black matter contained in the stomach was, in all probability, effused blood, altered by the acid.

3. *Experiment to shew the Effect of a large Dose of Corrosive Sublimate.*

A drachm of corrosive sublimate was introduced, as in the last experiment, into the stomach of a dog. The animal was greatly depressed, but remained tranquil, and exhibited little outward sign of pain. It was alive between four and five hours after the administration of the sublimate, but was found dead when seen afterwards.

*Dissection, 22 Hours after Death.*—The œsophagus was natural.

The stomach, externally, was highly vascular. It contained several ounces of a thin, dark-coloured fluid. The whole inner lining was of a leaden hue; this was the mucous membrane, which appeared to be universally in a state of slough, but it was in no part detached.

The duodenum, at its commencement, presented a mixed appearance, partly red, partly of a lead colour, as if the irritant and corrosive effects of the poison were here blended; the mucous membrane was thickened and had a roughened aspect.

The whole of the small intestines were inflamed, a thick white mucus being generally effused upon their mucous membrane.

Dr. Roupell remarks that, probably, the quantity of corrosive sublimate administered in this experiment, is greater than would be voluntarily or accidentally swallowed. From this circumstance, the morbid changes usually produced in the stomach resemble those observed in the œsophagus in this instance.

4. *Experiment to shew the Effect of Alcohol upon the Mucous Membrane.*

An ounce of rectified spirit, mixed with an ounce of water, was introduced into the stomach of a large dog. In a few minutes it ran about in a hurried manner, uttering a bellowing sound, and making attempts at vomiting. In ten minutes, it was "dead drunk." Next day it was so much recovered, that it was killed by a blow upon the head, and immediately examined.

The œsophagus was natural.

The stomach was slightly vascular externally, and presented a well-marked hour-glass contraction. It contained an ounce of a thin bloody fluid, mixed with frothy mucus. The rugæ were prominent, and those of the large extremity were almost universally covered with patches of ecchymosis of a deep crimson colour. The intervening portions of mucous membrane retaining their healthy appearance.

The more usual appearances, after taking spirits, says Dr. Roupell, are not such intense congestion as in this instance, but a dusky-red colour of the membranes. Cases, however, have occurred, where death has resulted from a large quantity of spirits swallowed at a draught, yet no obvious alteration of structure could be detected. In these instances, we may suppose that the action of the alcohol upon the nervous system has been so powerful as to destroy, before any local alterations were produced.

The experiment shews the mode in which the abuse of ardent spirits affects the mucous membrane of the stomach. The gin-drinker always suffers from chronic gastritis, and the mucous membrane is found on dissection thickened or softened from chronic inflammation. The liver incessantly stimulated, takes on a form of inflammatory action also, its acini become enlarged, and at last the tuberculated condition is produced. When to these more obvious and immediate consequences of the use of ardent spirits we add the frequent concomitants and sequelæ — the hypertrophic heart, the thickened tunics of the arteries, the consequent apoplexy or dropsy—or, in weaker constitutions, the phthisis, perhaps the malignant disease—we have pictured to ourselves the happy proselyte to the worship of Gin, his prostrate adorer in those whitened sepulchres, his modern temples. And this, as Benedict says, is our conclusion.

II. FRAGMENTA DE VIRIBUS MEDICAMENTORUM POSITIVIS, SIVE IN SANO CORPORE HUMANO OBSERVATIS.—A SAMUELE HAHNEMANN, M. D. &c. &c. &c. Edidit F. F. QUIN, M.D. &c. &c. &c. Londini. Veneunt apud S. Highley, MDCCCXXXIV.

This is altogether an extraordinary work, extraordinary at least considering that it is edited, printed, and published in this city of London, in the year of our Lord 1834. What physician, surgeon, or apothecary would dream of writing a book in Latin?—Who are to be the readers?—The profession? It will never peruse one page, unless some unhappy dog-eared critic shall deem himelf in duty bound to "pause and ponder and ponder and pause" at its curiously concocted paragraphs. If the faculty eschew the remarkable production, we are sure it will never find favour with the public. No general reader, in fact, could understand it. The whole affair is a phenomenon.

Quale portentum neque militaris
Daunia latis alit esculetis;
&c.

A clerical admirer of homœopathy has ventured to indulge in the auspicious prophecy, that the period will arrive when Hahnemann, "that young man," will come on us like Punch in all his glory. The dogmatists and the empirics, and the proselytes and professors of all other sects in the pantisocrasy of medicine, should make the most of the time they have, for their days assuredly are numbered. If prodigies and portents usher in revolutions and wait on the rise and the fall of dynasties—if Brutus cannot plant a dagger in Cæsar, without exciting the wrath of a comet, and turning the solar system topsy turvy—we may fairly suppose that the fall of the ancient science of medicine will excite some little pother in the land of spirits, and set those deities, or gnomes, or sylphs, or whoever else the scene-shifters may be, to contrive some omen or portentous accident, which may lead the most thoughtless to expect a change. Such an omen we consider this book. Good God! only to think of our publisher, Mr. Highley, being involved in the diablerie. He has certainly sold himself to Mephistopheles, and his family may find in some secret drawer a bond, apparently written, in red ink, but actually signed, oh horror! in his blood!—Poor Mr. Highley!

The charming or charmed pages (their number is exactly 212) are occupied with an enumeration of the mystic properties of certain chemicals or drugs, amongst which we may enumerate the aconitum napellus—arnica montana—atropa belladonna—chamomilla matricaria—cocculus menispermum—copaifera balsamum—digitalis purpurea—ipecacuanha—ledum palustre, and various other pharmaceutical simples—some of which are usually considered active, whilst others are commonly deemed inert in their effects.

Midas, we all know, had the faculty of turning what he touched to gold. We will not affirm, nor will we even hint, that Hahnemann or his disciples enjoy the power, nor express the wish to convert the cube root of rhubarb to a sovereign, or the infinitesimal extraction of the chamomilla matricaria to half a guinea neatly wrapped up in white paper. But we do maintain that the new doctrine is possessed of the marvellous property of making all it touches homœopathic—and that facts, conclusions, observation, experience—all become invested with new faces and new forms when fused in its alchemical alembic. We may take the instance of feverfew—matricaria chamomilla.

The carelessness and ignorance of the old system of physic condemned this potent remedy to banishment from the shelves of the chemist and apothecary. Its name was seldom seen but in treatises on botany, and its virtues were appreciated in the recipes of old women, rather than in the orthodox prescriptions of doctors. Yet the wand of Homœopathy has converted this desert to a garden, and the forsaken feverfew blushes to find herself diffused through thirteen goodly pages, and adorned with two hundred and seventy-two properties! The astonishment of the reader at the pleasing change resembles that of the hermit in the ballad, at recognizing in his guest a lovely maid.

The bashful look, the rising breast,
Alternate spread alarms;
The lovely stranger stands confest
A maid in all her charms.

Some of these properties, we allude to the feverfew and not to Angelina, are singular and unexpected. We shall signalise a few. The 29th runs thus:—

"Cogitationes, ideæ evanescentes."

The 45th requires that an infant should be dandled; for, a fact so remarkable, that, unless well attested, we could not have believed it, the baby grows restless if not in arms.

"Non nisi gestatus quiescere potest infans."

The 51st evinces the accurate observation of the homœopathists. A patient who imbibes the matricaria chamomilla grows sulky for exactly the period of two hours.

"Morositas per duas horas durans."

The 88th property particularizes a species of discriminating tooth-ache, which is probably familiar to the dentists. The singularity consists in the tooth contracting a violent antipathy to coffee.

"Odontalgia post haustum calidum (maximè coffeæ) potum sæviens."

But the 102d and 103d qualities of feverfew are more wonderful. Under its influence the teeth begin to elongate, like the faces of the professors of the old system of physic, and, what is more, they actually stagger.

"Dentes elongati."

"Dentium vacillatio."

We think it would be useless to add to the extraordinary catalogue. We assure our readers that these are not choice flowers, discovered with difficulty and selected with care. They came in our way, and so we plucked them; and any one who wanders through the thickly-planted pages of this curious work, may gather at each turn as extraordinary specimens. It would be idle to eulogize the facts of homœopathy; displaying as they do the brilliant imagination of the homœopathist: in point of ingenuity they appear to us to excel the doctrine. None can, of course, doubt that the new system will soon rise to strength and maturity on the ruins of the ancient errors, of experience, observation, and induction; and, selfish though it may appear, we congratulate ourselves on having discerned its lurking beauties and its infant vigour, at the time when the fools and bigots of the 19th century rejected and despised it.

THE PRINCIPLES OF DIAGNOSIS, Vol. I. Second Edition. By MARSHALL HALL, M.D. F.R.S. L. and E. etc. Octavo, pp. 426. London, 1834.

It is a pity that Dr. Hall has not mentioned in a preface or advertisement of some description, the alterations, additions, or reductions he has made in the present edition. Harassed and perplexed as the critic is by the claims of authors and the multiplicity of books, he cannot be expected to compare editions, and to extricate a few separate grains of novelty from bushels of familiar matter. We would recommend those gentlemen whose merits or whose fortune convey them past the rubicon of a first edition, to signify the changes that occur in all successive ones.

In the present instance we are prevented from doing adequate justice to Dr. Marshall Hall, by our inability to inform our readers of the real quantity of added matter or improved arrangement. But the fact of a second edition being called for, is itself a high compliment to the author and the work, as the latter is one which, for obvious reasons, can only be purchased and appreciated by the profession.

We have on various occasions endeavoured to do justice to the industry and the talent of Dr. Marshall Hall. Indeed, our previous notice of his labours was so full as to prevent us from doing much more than mention the appearance of this new edition, and recommend those to purchase it immediately who have not the advantage of possessing the first. We may glance at Dr. Hall's arrangement before we quit the subject. The work contains four sections. The first is occupied with the history of diseases—the second is devoted to their symptoms—the third is occupied with the effects of remedies—and the fourth is appropriated to their morbid anatomy. The second section is, of course, the longest. It treats in order of the morbid appearances of the countenance—the morbid conditions of the attitude—the morbid appearances of the tongue, &c.—the morbid conditions of the general surface—of some morbid conditions of the general system—of the morbid states of the functions of the nervous system—of the morbid affections of the function of respiration—of the morbid affections of the circulation—of the physical conditions of the thorax—of the functional affections of the alimentary canal—of the functional affections of the urinary organs—of the physical conditions of the abdomen.

Such is our author's bill of fare, and as we cannot praise particular dishes, we recommend the whole. Our readers may taste and judge for themselves. By the way, we may mention that the doctor is about to give oral utterance to these principles of diagnosis. In other words, he is the future lecturer on the practice of physic at the Aldersgate Street Medical School. We wish him all the success he deserves, and that is not a trifle.

IV. A Demonstration of the Nerves of the Human Body. By Joseph Swan. Quarto, pp. 82. Plates, XXV.

We have drawn attention to Mr. Swan's folio plates of the nerves. The four fasciculi of that splendid work do honor to Mr. Swan, and are creditable to the country. The laborious industry that executed the dissections, the patient zeal that directed and superintended the artists, and the graphic powers of the latter, have combined to produce a work unequalled in any period of the history of anatomy for its accuracy and its beauty. Cheap though it is, the actual price must prevent the great majority of the profession from buying it. Young men who want it most, can afford it least, and the older and wealthier members of the profession who are able to afford it, will not, perhaps, be persuaded that they want it. We fear then that the sale of the folio copy will never be extensive.

But the quantity of trashy anatomical plates which fill the market and disappoint and deceive the purchaser, renders it a matter of high importance to circulate good ones at the lowest possible price. Actuated by this laudable desire, Mr. Swan has now published in a quarto form the plates which are contained in the folio edition. The accuracy of the dissections, and the beauty of the drawings continue unimpaired—the diminished size is scarcely attended with diminished clearness—and the moderate price will enable students of indifferent means to purchase what is really worth their money and their time, instead of unprofitably consuming both in the acquisition and the study of what are neither decent drawings nor accurate charts.

We recommend all and every gentleman desirous of becoming perfectly acquainted with the anatomy of the nervous system, to make himself master of one or other form of these Demonstrations. If he does not enjoy a superfluity of cash, let him stint himself in some less important matter, and parents or guardians may well expend a pound or two in placing the work in the hands of the young aspirant for anatomical distinction.

V. An Essay on the Deaf and Dumb, &c. &c. Second edition. By John Harrison Curtis, Esq. &c. 1834.

We have heard on good authority that in the instances of more than one individual, considered as irremediably deaf and dumb, Mr. Curtis has by strict attention and judicious treatment removed the deafness, and, of course, the dumbness. The facts which have come to our knowledge induce us to recommend practitioners to attend to the suggestions of Mr. Curtis, and in cases of this nature to pause before they doom to a sort of civil banishment, unhappy individuals whom cautious observation and appropriate management might restore to the advantages and pleasures of society. We have only time at present to direct attention to some of the details of Mr. Curtis' work, and to express our opinion that his cases and suggestions deserve consideration.

VI. A Practical Treatise on Lepra Vulgaris, with Cases that have uniformly yielded to the Plan of Treatment laid down, &c. &c. By Edward Beck, M.D. Ipswich. Octavo, pp. 74.

The object of Dr. Beck is to display the obstinacy of lepra and psoriasis—their indisposition to yield to the treatment usually recommended—and the superior efficacy of the plan which he proposes and pursues. Without further preface, we shall state the plan in question.

" When the disease is of considerable standing and attended with much pain, soreness, and inflammation of any one part, a preparatory plan of treatment will be required, which is to be principally antiphlogistic.

I have generally recommended abstinence from wine, and prescribed a few doses of Aperient Medicine: equal parts of Pil. Rhei Comp., and Extr. Colocynth Comp. with or without two or three grains of Pil. Hydr. answer the purpose well. The Sulph. Precip., in combination with Sod. Subcarb. Exsic. in doses of half a drachm of the former, to 5 grs. of the latter, two or three times in the day, so as to act freely on the bowels, has a beneficial influence in reducing the irritation. The Liq. Plumb. Subacet. Dilut., or a lotion containing the Plumb. Subacet. in combination with Zinci Sulphas, I have found the best local applications, during the very irritable state of the disease: two scruples of the former, with half a drachm of the latter, to a pint of Distilled Water."

The preparatory plan, therefore, consists of active aperients for some days—the application of the lotion that is found to suit best—and, when much local irritation exists, the use of the powder of sulphur and soda three or four times during the day. Sometimes there is no necessity for a preparatory plan of this description. As soon as the irritation and inflammation of the parts are moderated, the specific treatment is required.

" The specific plan consists in applying the following liniment to the parts affected :—

℞.—Picis Liquid.
Sulphuris.
Adipis Præpar. sing. unciam misce.

The following pills are at the same time to be taken regularly :—

℞.—Picis Liquidæ unciam dimidiam. Flor Tritici q. s. misce. et divide in Pilul. sing. gr. v.—iii. ad vi. ter in die sumantur.

Where the irritation is very considerable, a milder liniment may first be had recourse to, as the following :—

℞.—Picis Liquidæ.
Sulphuris singul. unciam dimidiam.
Adipis Præparat. unciam. misce.

This preparation, should it irritate very much, may be gently rubbed on the parts affected with the Leprous eruption at night, allowed to remain on a few minutes only, and then wiped off with soft linen. The application usually increases the pain for a few minutes, but afterwards the parts become much more comfortable to the Patient's feelings. In three or four days the former liniment can be borne and will be required, and it may be allowed to remain on the part all night, and be washed off in the morning, or it may be applied and remain on for days together, for it is better that the part should not be frequently washed, as the local irritation is considerably increased thereby. Where a large surface, a limb for instance, is covered with the Leprous eruption, it may be gently rubbed well over with the liniment, and the part covered with linen (in the form of a stocking if it be the leg, or a glove if it be the fore-arm, or back of the hand.) Every second or third day will suffice to apply the liniment afresh. This is a very efficacious mode of applying the liniment, but is adapted only for those in the lower class of society, who might have no objection to the scent of the ointment. Among the upper class of society, this plan might be seriously objected to, in which case the application should be used at night only, and washed off in the morning. But where single patches are situated, some of the ointment should be applied thickly to them, and two or three pieces of lint confined over it, with strips of adhesive plaster—this may be allowed to remain for days together, and no scent can escape if the lint be well confined. Most of the small Leprous spots, which are not of long duration, will disappear under the general plan of treatment, and only an occasional large patch will require this attention for a short time.

As the Pills sometimes occasion head ache and disagree with the stomach at first, they may be commenced with by taking two, three times in the day, and very gradually increased to six, three times in the day, which will be found an efficient dose, and as much as most stomachs will bear well. The torpid state of bowels that almost invariably accompanies Lepra, usually gives way to the use of the Pills.

Where the disease is of short duration, and is not confluent, the preparatory plan of treatment may, in a great measure, be dispensed with, both locally and generally.

A single dose of the Pills at night, followed in the morning by a tea-spoonful or two of calcined Magnesia, or half an ounce of the sulphate, will be found sufficient; but, without some preparation for the Pilulæ Picis, they will be found to occasion nausea and head-ache, at which the patient, taking an early disgust, will, with difficulty, be persuaded to resume the use of them. In all cases, where the local irritation is considerable, the stronger form of the ointment may, from the first, be had recourse to. After the patient is able to take a full dose of the pills, the use of aperients may, in almost every instance, be discontinued, for the Pil. Picis alone regularly and sufficiently act upon the bowels.

I particularly wish to point out the necessity, where much local irritation is present, of using the milder Ung. Picis Comp., and that it remain on only a minute or two. Without this precaution, the patient may take a prejudice against the application, from the increase of pain it produces, and discontinue it altogether."

Both pills and ointment are necessary to effect a cure, and their use should be continued for some time after the disappearance of the disease. Two months, or even more, will be required for the cure of severe or long-continued lepra.

Our author's treatment of psoriasis is put forward with less confidence than that of lepra. It consists of antiphlogistic treatment while heat and irritation are considerable—aperients—after a few days the application of an ointment, consisting of half an ounce of camphor, and two ounces of cerat. cetacei—and the exhibition of sulphur and soda.

We have no space left for comments, which we leave to the fancy, the judgment, or experience of our readers.

